# Identification of new components of the RipC-FtsEX cell separation pathway of *Corynebacterineae*

**DOI:** 10.1371/journal.pgen.1008284

**Published:** 2019-08-22

**Authors:** Hoong Chuin Lim, Joel W. Sher, Frances P. Rodriguez-Rivera, Coralie Fumeaux, Carolyn R. Bertozzi, Thomas G. Bernhardt

**Affiliations:** 1 Department of Microbiology, Blavatnik Institute, Harvard Medical School, Boston, Massachusetts, United States of America; 2 Department of Chemistry, Stanford University, Stanford, California, United States of America; 3 Howard Hughes Medical Institute, Chevy Chase, Maryland, United States of America; Ludwig-Maximilians-University Munich, GERMANY

## Abstract

Several important human pathogens are represented in the Corynebacterineae suborder, including *Mycobacterium tuberculosis* and *Corynebacterium diphtheriae*. These bacteria are surrounded by a multilayered cell envelope composed of a cytoplasmic membrane, a peptidoglycan (PG) cell wall, a second polysaccharide layer called the arabinogalactan (AG), and finally an outer membrane-like layer made of mycolic acids. Several anti-tuberculosis drugs target the biogenesis of this complex envelope, but their efficacy is declining due to resistance. New therapies are therefore needed to treat diseases caused by these organisms, and a better understanding of the mechanisms of envelope assembly should aid in their discovery. To this end, we generated the first high-density library of transposon insertion mutants in the model organism *C*. *glutamicum*. Transposon-sequencing was then used to define its essential gene set and identify loci that, when inactivated, confer hypersensitivity to ethambutol (EMB), a drug that targets AG biogenesis. Among the EMB^s^ loci were genes encoding RipC and the FtsEX complex, a PG cleaving enzyme required for proper cell division and its predicted regulator, respectively. Inactivation of the conserved *steAB* genes (cgp_1603–1604) was also found to confer EMB hypersensitivity and cell division defects. A combination of quantitative microscopy, mutational analysis, and interaction studies indicate that SteA and SteB form a complex that localizes to the cytokinetic ring to promote cell separation by RipC-FtsEX and may coordinate its PG remodeling activity with the biogenesis of other envelope layers during cell division.

## Author summary

The pathways involved in bacterial surface assembly are critical for cell morphogenesis and serve as attractive targets for antibiotic development. Bacteria in the suborder Corynebacterineae, which includes important pathogens like *Mycobacterium tuberculosis*, possess a unique multilayered surface structure. In addition to the common peptidoglycan cell wall, they have an attached polysaccharide layer called arabinogalactan and an outer membrane made of mycolic acids. To enhance our understanding of cell surface biogenesis in these bacteria, we performed a global genetic analysis of gene function in the model system *Corynebacterium glutamicum* (*Cglu*) using transposon sequencing. In addition to defining the essential gene set in this organism, our analysis also identified SteA and SteB as components of the cytokinetic ring. These factors are conserved among the Corynebacterineae, and our results reveal that they play a critical role in the final stages of cytokinesis by promoting remodeling of the peptidoglycan layer at the division site.

## Introduction

**Fig 1:**
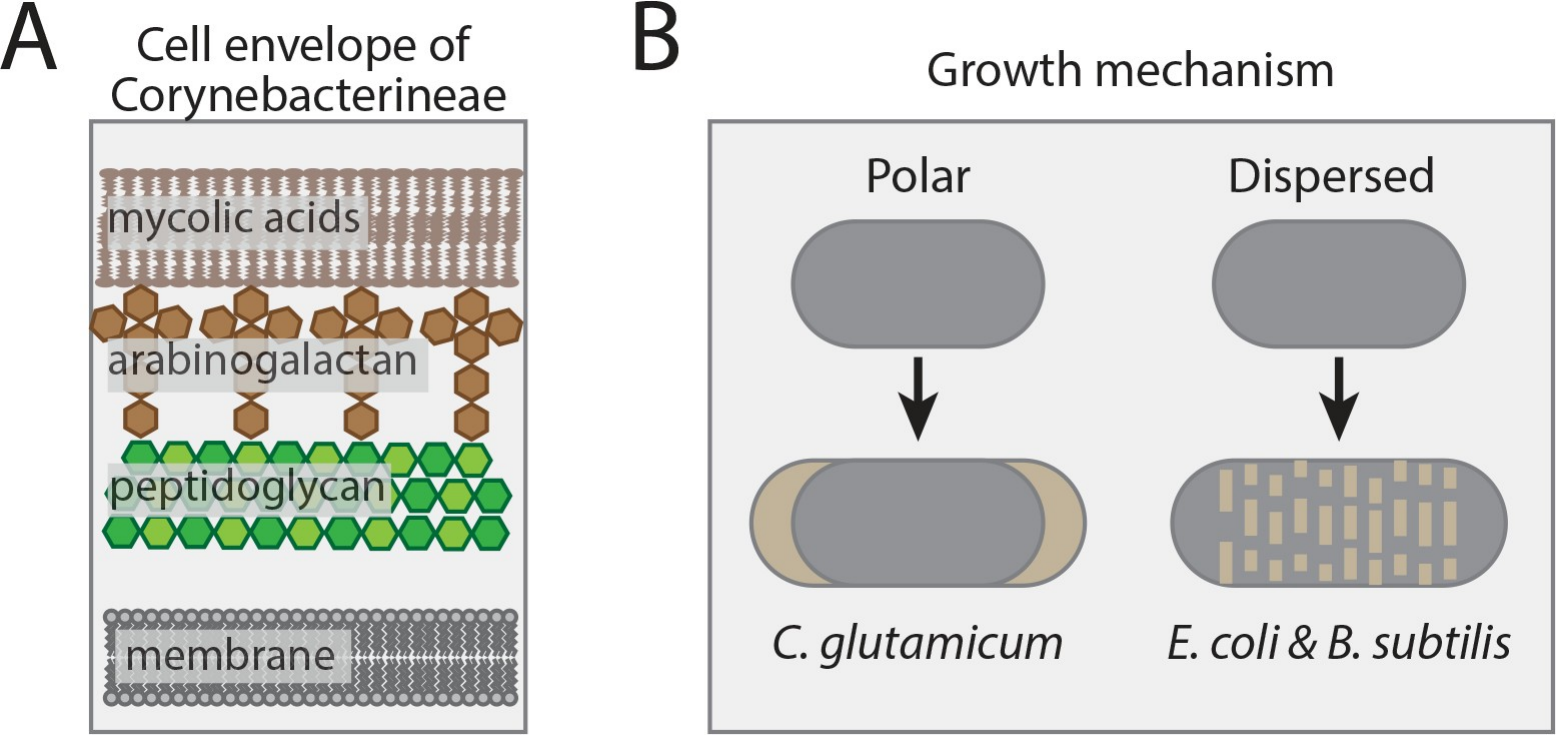
Cell envelope architecture and growth mechanism of Corynebacterineae. (**A**) Shown is a schematic of the unique triple-layered cell envelope structure of bacteria in the Corynebacterineae suborder. (**B**) Comparison of the growth modes of *C*. *glutamicum* and other mycolata relative to that of the model organisms *E*. *coli* and *B*. *subtilis*. Grey: old cell wall material; tan: nascent cell wall material.

The Corynebacterineae suborder of bacteria includes many significant human pathogens, including *Mycobacterium tuberculosis* (*Mtb*) and *Corynebacterium diphtheriae* [1]. These organisms are often referred to as the mycolata due to the distinct architecture of the cell envelope that surrounds them. Their mode of growth also differs substantially from other well-studied bacterial model systems such as *Escherichia coli* and *Bacillus subtilis*. Like the traditional model bacteria, members of the Corynebacterineae have a cytoplasmic membrane that is surrounded by a crosslinked polysaccharide cell wall layer made of peptidoglycan (PG). However, their PG matrix is uniquely modified with a second polysaccharide polymer called arabinogalactan (AG), which serves as the anchor point for long hydrocarbon chains of mycolic acids (MA). Together with other free mycolate glycolipids, the anchored MAs form an outer membrane-like layer called the mycomembrane that is reminiscent of the second membrane of Gram-negative bacteria (**Fig 1A**) [2,3]. With respect to growth mode, bacteria in the Corynebacterineae suborder elongate by adding new cell envelope material at the cell poles rather than at dispersed sites throughout the cell cylinder like *E*. *coli* and *B*. *subtilis* (**Fig 1B**).

Although many of the enzymatic steps required for the synthesis of envelope components have been defined in the Corynebacterineae [4], it remains unclear how the biogenesis of the different layers is coordinated during growth and cell division. The mechanism of polar growth also remains poorly defined. Enhancing our understanding of these processes will address fundamental unsolved questions in microbiology while also providing information that is relevant to the treatment of diseases caused by mycobacteria and other Corynebacterineae. Drugs that target the assembly of the envelope feature prominently in the current therapeutic regimen for *M*. *tuberculosis* infections [5]. However, the rise of resistant organisms is eroding the efficacy of these drugs such that new classes of antibiotics are needed [5]. Given its status as a proven target, gaining new insights into the biogenesis of the mycolata cell envelope will provide the knowhow necessary for discovery programs to effectively develop next-generation therapies.

The non-pathogenic bacterium *Corynebacterium glutamicum* (*Cglu*) has been a useful model organism for mechanistic studies of mycolata envelope assembly [3,4]. Relative to the major mycobacterial model *Mycobacterium smegmatis* (*Msmeg*), it has a faster doubling time (1 vs. 3 hr) and a more compact genome (3.3 vs. 7.0 Mbp) that is likely to reduce the incidence of redundancy and aid genetic analyses. Another advantage of *Cglu* is that it tolerates more severe defects in the cell envelope, allowing the construction of mutants defective for AG and/or MA synthesis that have provided key insights into the biogenesis pathways for these envelope layers [6–9]. Where studies of *Cglu* have lagged behind those of the related mycobacteria is in the area of global genetic analyses. Only one large-scale transposon mutagenesis study was performed in *Cglu* thus far [10]. However, the library constructed only contained on the order of 11,000 mapped insertion mutants. We therefore set out to generate the first high-density library of transposon insertion mutants (>100K insertions) in this organism to enable transposon-sequencing (Tn-Seq) studies [11] for the discovery of new factors involved in envelope assembly in *Cglu* and other members of the Corynebacterineae.

Sequencing of the transposon library following growth in rich medium allowed the identification of the essential gene set of *Cglu*, which will provide an important resource for the community. To begin identifying novel envelope assembly proteins, the insertion profile of the library was also analyzed following growth in the presence of sub-lethal concentrations of ethambutol (EMB), a drug that disrupts AG biogenesis [12,13]. Several genes required for normal tolerance to EMB were identified using this chemical genetic approach, including the cell division genes *ripC*, *ftsE*, and *ftsX*. A similar phenotype was observed for genes of unknown function, which we have designated as *ste* loci (sensitive-to-ethambutol). Among these genes, mutants inactivated for the *steA* (cgp_1603) and/or *steB* (cgp_1604) genes were found to have cell separation defects similar to those of *ripC* and *ftsEX* mutants. Using a combination of quantitative microscopy, mutational analysis, and interaction studies, we discovered that SteA and SteB form a complex that localizes to the cytokinetic ring to promote cell separation by the RipC-FtsEX complex and may coordinate its PG remodeling activity with the biogenesis of other envelope layers during cell division. The success of this initial study suggests that further global genetic analyses in *Cglu* will provide a rich discovery platform for generating insights into the processes of cell growth and envelope biogenesis among the Corynebacterineae.

## Results

### Transposon mutagenesis and sequencing in *Cglu*

**Fig 2:**
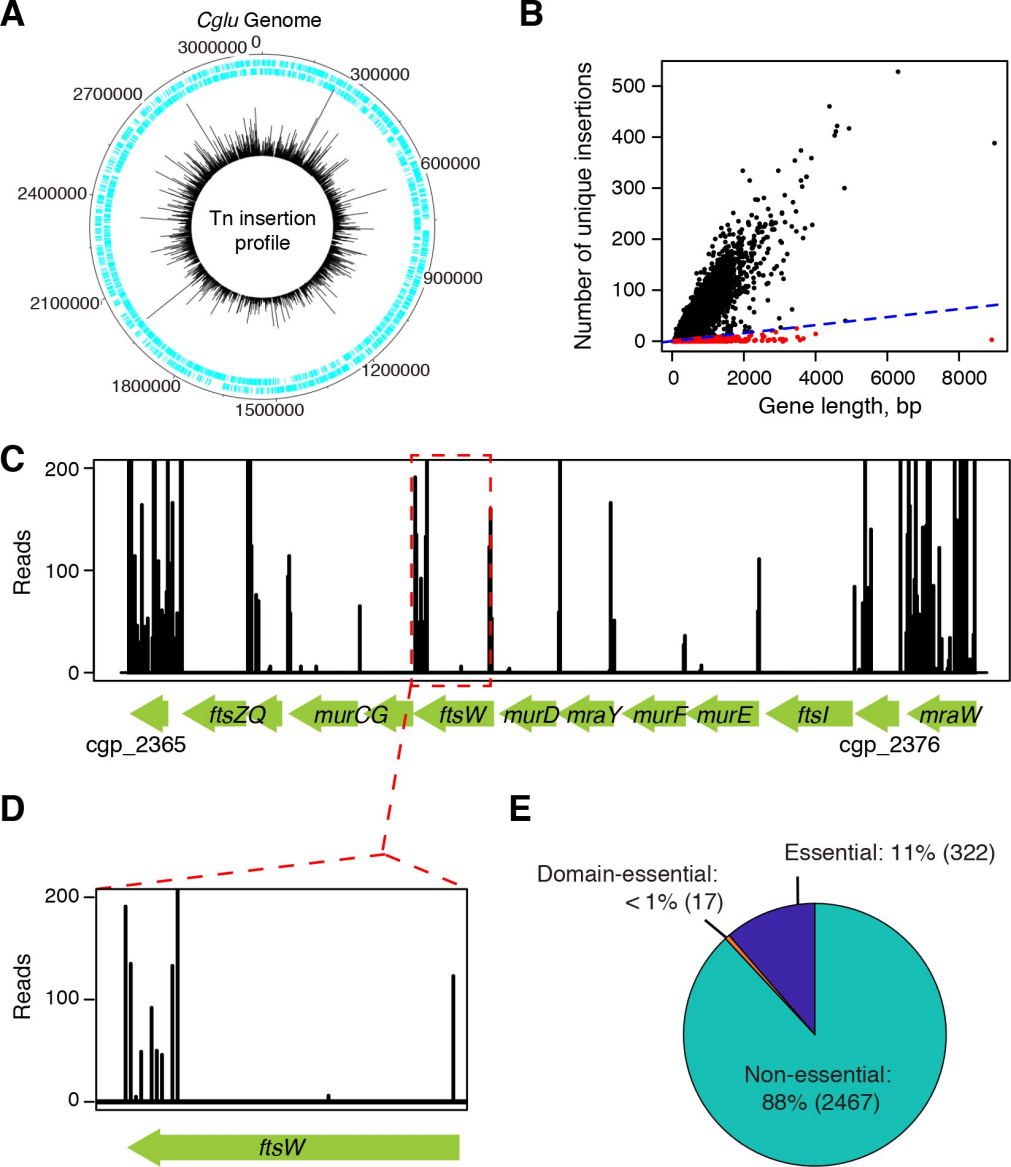
Overview of the Tn-seq analysis. (**A**) Location of all transposon insertions mapping to the *Cglu* MB001 genome. The cyan lines represent the location of each gene on the genome. The black bars represent the location of each transposon insertion with the height of each bar representing the detection frequency of each insertion. The numbers are the genome coordinates in base pairs. (**B**) The number of unique transposon insertions mapped to every gene of MB001 plotted as a function of gene length. For non-essential genes (black dots), the number of unique transposon insertions mapped linearly correlates with gene length. Genes that fall below the blue dashed line (red dots) have two-standard-deviations fewer unique transposon insertions than the mean value and therefore are designated as essential genes. (**C**) View of the Tn-Seq data for the highly conserved *dcw* locus encoding many essential genes. (**D**) The *ftsW* gene is shown as an example of Domain Essential (DE) gene. DE genes are genes that tolerate transposon insertions in some portion of their the 3’ ends but are depleted of insertions at the 5’ end. (**E**) Pie chart depicting the breakdown of essential, non-essential and DE genes categorized by the Tn-Seq analysis. Numbers in parenthesis correspond to the number of genes in the specified category.

The MB001 strain of *Cglu* was chosen for this analysis. It is a commonly used derivative of ATCC 13032 that has been engineered to remove three defective prophage elements [14]. We used the Tn5-based transposome system for transposon mutagenesis to yield a library of approximately 200,000 mutant colonies. Sequencing the transposon-chromosome junctions in the library yielded 10,419,920 reads that mapped to 200,940 unique insertions sites throughout the genome (**Fig 2A**). This high-density insertion map allowed the global identification of essential genes in *Cglu*. Such genes were identified by plotting the number of unique insertions per annotated gene relative to gene length (**Fig 2B**). The analysis revealed a bimodal distribution with the majority of genes displaying an increasing number of insertions proportional to their length and a small subset of genes that harbored very few insertions regardless of their size (**Figs 2B and S1**). Genes likely to be essential for growth in rich medium were defined as those harboring one or fewer unique insertions per 127 bp. With this cutoff, the percentage of essential genes was constant across a broad-range of gene lengths except for those shorter than 100bp (**S1 Fig**). For these smaller genes, an essentiality designation likely reflects technical factors of gene size and transposon density rather than biological function. We therefore excluded genes shorter than 100 bp from the final essential gene list along with tRNAs, rRNAs, and insertion elements. With these exclusions (131 in all, **S1 Table**), a total of 322 essential genes were identified in *Cglu* (**S1 Table**), which corresponds to roughly 11% of the total gene content, a percentage that is in-line with similar analyses of other bacteria, including *Mtb* [15–18]. Of the 490 total essential genes in *Mtb*, 445 have homologs in *Cglu*. However, only roughly half (231/445) of these are essential in *Cglu* (**S2 Table**). Thus, consistent with the conditional dispensability of the arabinan component of the AG and the MA layer of the envelope [6–9], *Cglu* is more tolerant to mutations and therefore should broadly enable studies aimed at defining the biological function of essential *Mtb* genes.

Support for the validity of our essential gene designations came from the fact that many genes expected to play vital roles in bacterial physiology were identified as such. For example, widely conserved genes in the division-cell wall (*dcw*) cluster with known essential roles in PG precursor biosynthesis (*murC*, *murD*, *murE*, *murF* and *mraY*) or cell division (*divIC*, *ftsI*, *ftsQ* and *ftsZ*) were found in to be depleted of transposon insertions in the profiling data (**Fig 2C**). Surprisingly, the Tn-Seq data indicated that *ftsW*, which encodes a cell division PG polymerase [19] essential for the growth of most bacteria, had a high number of insertions mapping within the reading frame, suggesting that it may not be essential in *Cglu*. Upon closer inspection, all of the insertions were found to be located in the 3’ end of the gene (**Fig 2D**). Notably, FtsW in *Cglu* and other Corynebacterineae possesses an extended C-terminal sequence absent from bacteria such as *E*. coli *and B*. *subtilis* (**S2 Fig**). In mycobacteria, this positively charged tail was previously shown to bind FtsZ and proposed to regulate its assembly into the Z-ring [20,21], the key cytoskeletal structure required for cell division [22]. Our results suggest that the FtsW-FtsZ interaction may not be essential for the viability of *Cglu*. Based upon the example of *ftsW*, we computationally searched for genes in which only 5’ end of the reading frame was essential. This analysis led to the identification of 17 “domain essential” genes (**S1 Table**) bringing the total essential gene content of *Cglu* to 339 (**Fig 2E**).

### Identification of ethambutol responsive loci

Ethambutol (EMB) inhibits the function of arabinosyl transferases responsible for the polymerization of arabinan chain of AG [12,13] as well as lipoarabinomannan (LAM), an arabinan-containing glycolipid found in the envelope of the Corynebacterineae [23,24]. Recent work has found that EMB treatment inhibits polar growth in *Cglu* and mycobacteria and causes defects in daughter cell separation [25,26]. We therefore reasoned that new factors involved in growth or division might be identified as loci causing EMB hypersensitivity when inactivated. To this end, the *Cglu* transposon library was propagated in the presence of a sublethal concentration of EMB (0.3 μg/ml) and then analyzed by transposon sequencing. Loci associated with sensitivity to ethambutol (*ste* genes) were then identified as those displaying a significant reduction in mapped insertions following EMB treatment relative to an untreated control.

**Fig 3:**
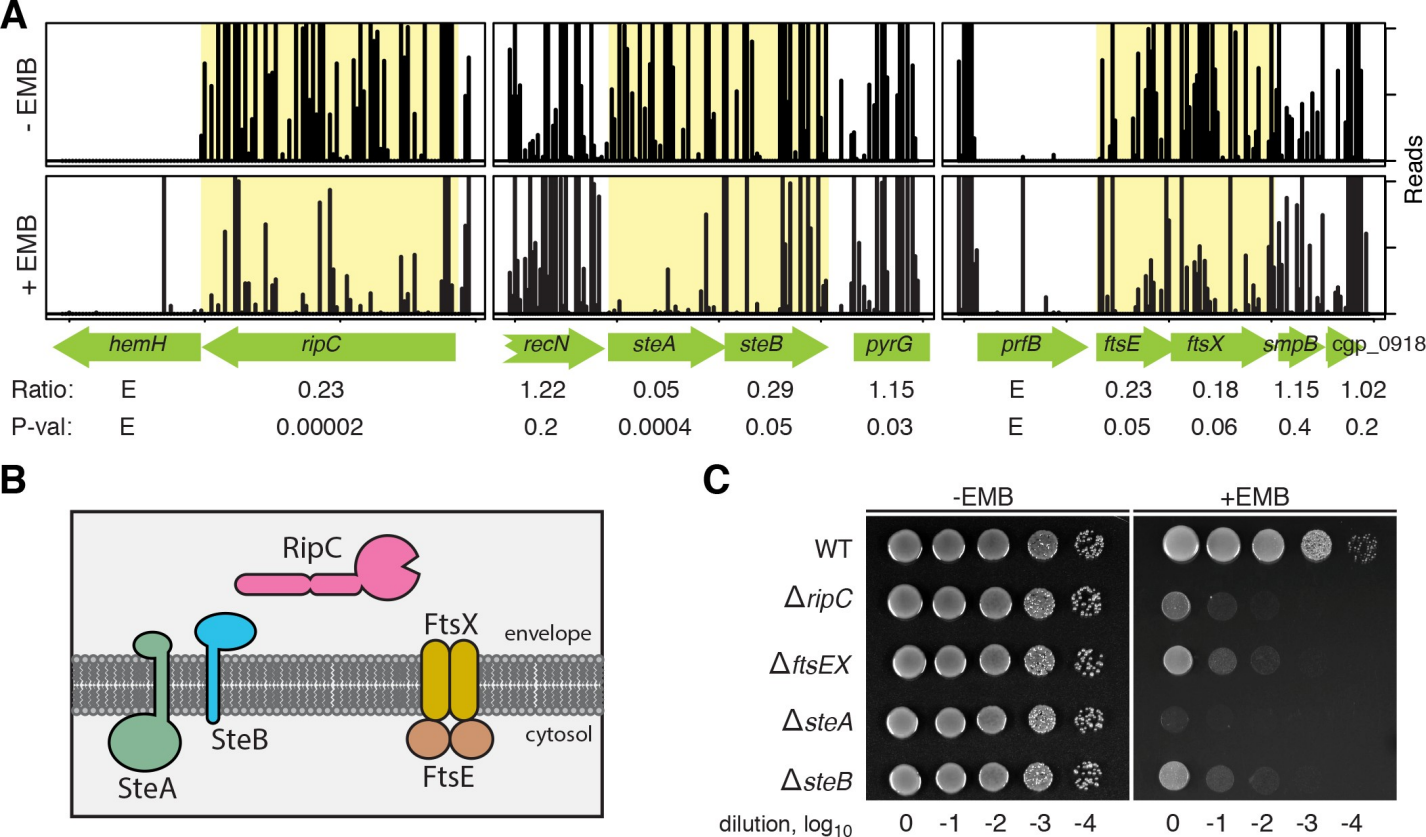
Identification of *s*ensitive *t*o *e*thambutol (*ste*) mutants by Tn-seq. (**A**) Shown are transposon insertion profiles for selected regions of the chromosome after growth in medium without and with EMB as indicated. The height of each line in the profile represents the number of sequencing reads corresponding to a transposon insertion at the indicated genome position. The ratio listed below each gene corresponds to the ratio of the total number of sequencing reads for all insertions in a given gene following growth in medium with EMB relative to the no drug control. P-value reports the statistical significance of the difference in Tn-Seq profiles for each condition based on the Mann-Whitney U test. Profiles highlighted in yellow correspond to genes that showed a statistically significant reduction in transposon reads following EMB treatment (ratio <0.33 and *p*-val <0.05). (**B**) Schematic showing the envelope localization and membrane topology of factors implicated in cell separation. The TMHMM algorithm predicts that both SteA and SteB have an N-in/C-out topology. (**C**) Overnight cultures of MB001 (WT) and its indicated deletion derivatives were normalized to an OD_600_ of 0.5, serially diluted, and spotted (5 μl) onto BHI agar medium with and without 1 μg/ml EMB as indicated. Plates were incubated for 24 hours at 30°C and photographed. Note that mutants with cell separation defects that form aggregates were vortexed for 3 seconds to resuspend the cells before OD_600_ measurements were taken for normalization.

A total of 49 *ste* loci were identified in the Tn-Seq analysis (**Figs 3A and S3A and S3 Table**). As an indication that the screen worked as expected, several genes encoding enzymes in the AG biogenesis pathway were identified as hits along with other loci encoding factors with predicted roles in envelope biogenesis, including two loci implicated in daughter cell separation: *cgp_1735* and *ftsE-ftsX*. The *cgp_1735* gene, which we will refer to as *ripC*, is homologous to a gene of the same name in mycobacteria and encodes an exported protein with an N-terminal coiled-coil domain and a C-terminal PG endopeptidase domain that cuts crosslinks in the cell wall matrix (**Fig 3A and 3B**) [27]. The *ftsE* and *ftsX* genes encode an ABC-transporter-like complex (FtsEX) (**Fig 3A and 3B**) that was originally implicated in the control of cell wall hydrolase activity in *E*. *coli* [28] and *Streptococcus pneumoniae* [29]. FtsEX was subsequently found to be involved in the activation of PG hydrolase activity in a number of organisms, including mycobacteria [30], and was recently suggested to function in complex with RipC to promote cell separation in *Cglu* [31].

**Fig 4:**
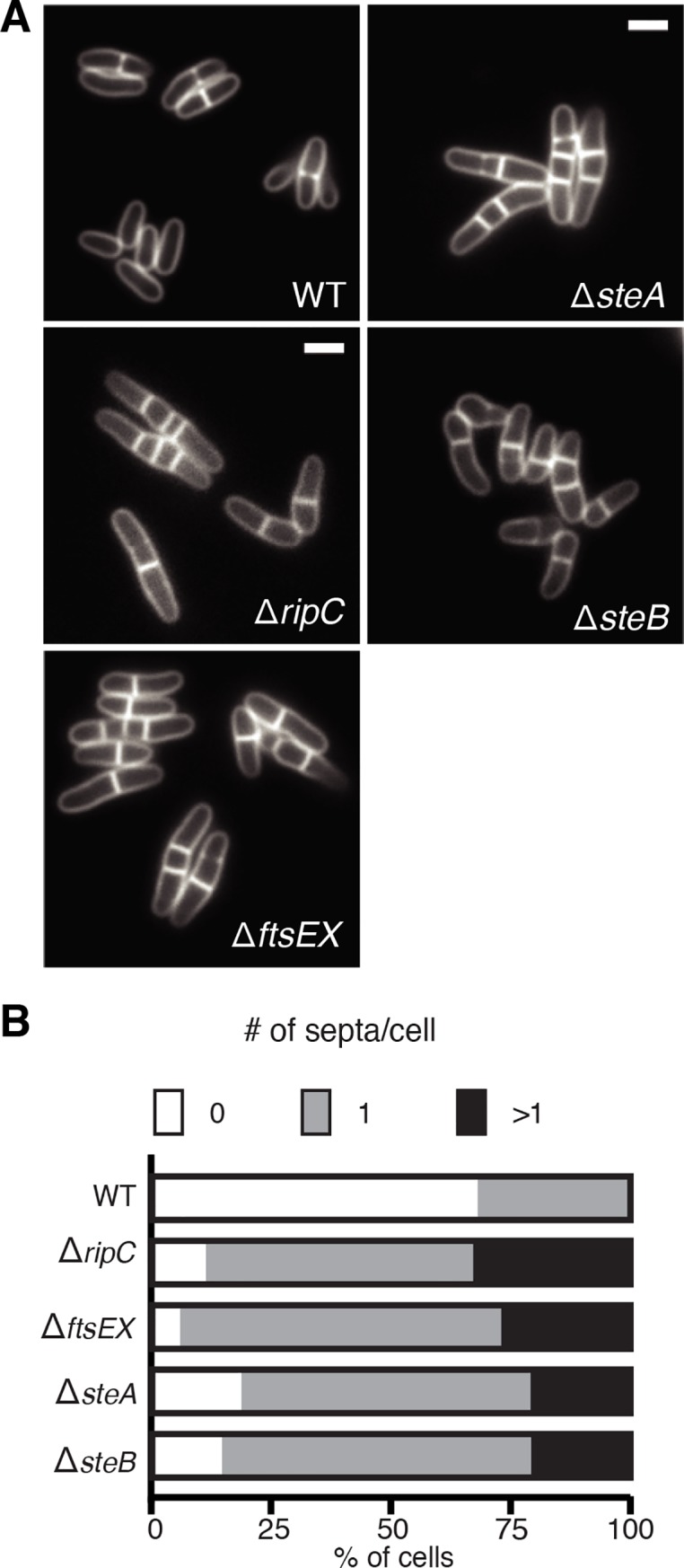
Cell separation defect of *ste* mutants. (A) Overnight cultures of MB001 (WT) and its indicated derivatives were diluted 1:1000 in BHI and grown at 30°C. When the OD_600_ of the cultures reached 0.2, cells were stained with FM 4–64 (1.5 μg/ml) for 2 minutes before they were spotted on agarose pads for visualization by fluorescence microscopy. Scar bar: 2 μm. (**B**) Assessment of cell separation defects by quantification of septa/cell. Overnight cultures of the strains from (A) were diluted 1:1000 in BHI and grown at 30°C. When the OD_600_ reached 0.2, cells were gently pelleted, resuspended and diluted 1:10 in BHI supplemented with the fluorescent D-amino acid HADA (1 μM). Following growth at 30°C for 3 hours, labeled cells were loaded on a CellASIC ONIX microfluidic plate, flushed with BHI and imaged by fluorescence microscopy. The number of septa per cell was tallied using the Cell Counter plugin in FIJI (*n* > 300 for each strain).

To validate the screening results, deletion alleles for several of the identified *ste* genes were constructed and the corresponding mutants were tested for EMB hypersensitivity. All of the deletion mutants constructed were found to display some degree of sensitivity to EMB (**Figs 3C, S3B**), indicating that the screen was faithfully identifying loci responsive to EMB. The morphology of each mutant was also assessed by fluorescence microscopy following staining with the membrane dye FM4-64. The number of septa per cell was also quantified following growth in the presence of a fluorescent D-amino acid (FDAA), which labels septa and other sites of new cell wall synthesis [32]. Indicative of their expected cell separation defect [26,27,31], mutants deleted for *ripC* or the *ftsEX* locus yielded a population of cells that were longer than average relative to wild-type and possessed one or more unresolved septum (**Fig 4A and 4B**). As previously observed [26], cellular compartments that were ‘trapped’ in the middle of chaining cells failed to elongate properly, presumably because their cell poles remained attached to the neighboring cells. Among the other *ste* mutants constructed, only deletions in *steA* and *steB* were found to cause a cell separation defect that resembled cells inactivated for *ripC* or *ftsEX* (**Fig 4A and 4B**).

The *steA* and *steB* genes are located in an apparent operon that is highly conserved within the Corynebacteriales order (**S4A Fig**). SteA is a predicted to be a bitopic transmembrane protein with an N-terminal cytoplasmic domain and a small C-terminal domain at the membrane surface (**Fig 3B**). SteB is also a single pass transmembrane protein, but it is predicted to have a small N-terminal tail in the cytoplasm and a soluble C-terminal domain exposed to the envelope (**Fig 3B**). As expected from the Tn-Seq data where *steA* displayed a greater depletion of transposon insertions following EMB treatment than *steB* (**Fig 3A**), a Δ*steA* mutant was found to have a more severe EMB-sensitivity phenotype than a Δ*steB* mutant. The double Δ*steAB* mutant was as sensitive to EMB as a single Δ*steA* mutant, indicating that *steA* is epistatic to *steB* (**S5 Fig**). Importantly, the phenotypes of all *steA/B* deletion mutants could be complemented upon ectopic expression of the missing gene(s), indicating that the phenotypes of the deletions were not due to adverse effects on the expression of nearby genes (**S5 Fig**). Given the importance of SteA and SteB for cell division and their conservation among the Corynebacterineae, we chose to investigate their functions further. Studies of the other *ste* loci will be pursued as part of a separate line of investigation.

### Genetic evidence that SteA and SteB function in the RipC cell separation pathway

**Fig 5:**
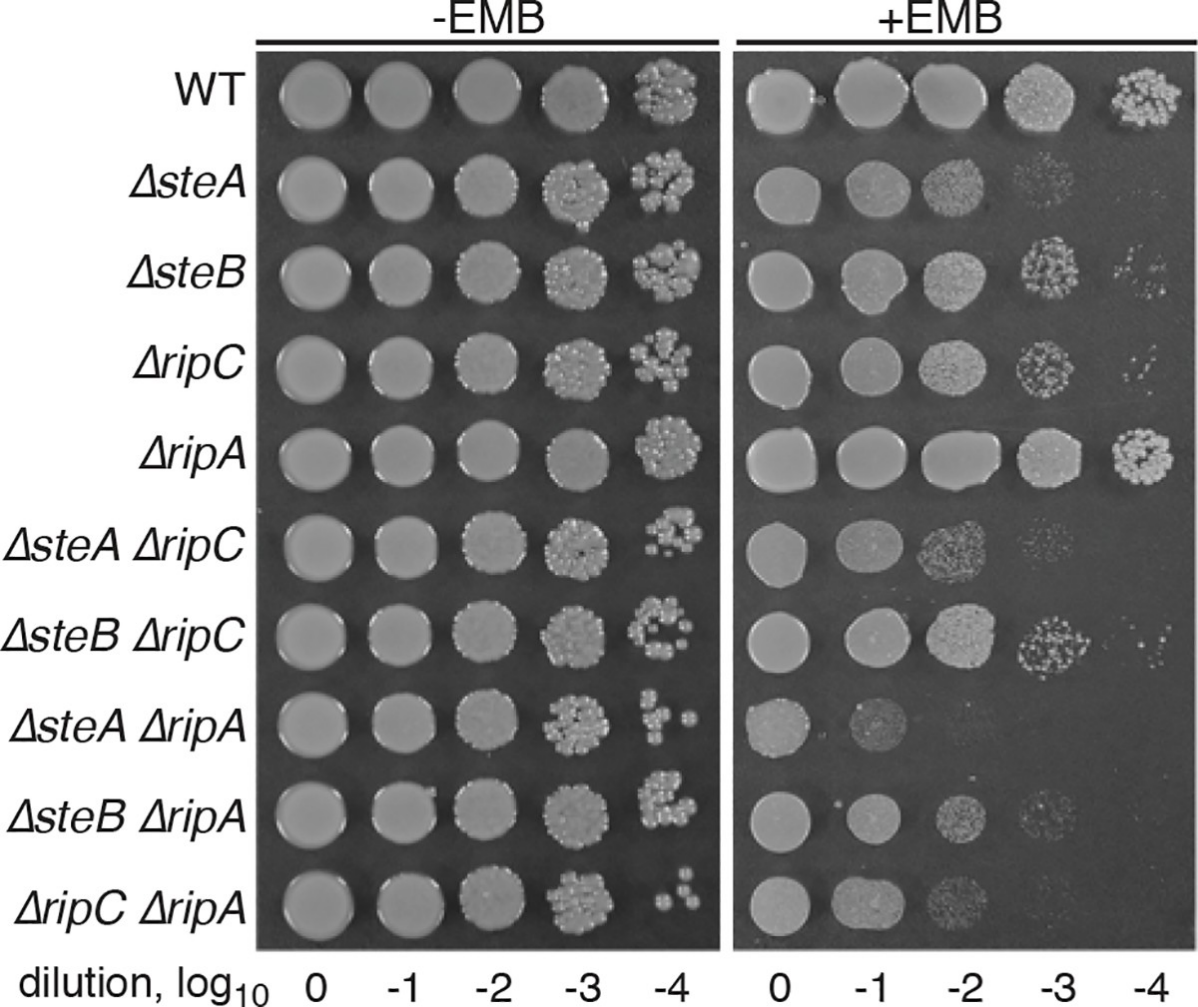
EMB sensitivity of double mutant strains. Overnight cultures of MB001(WT) and its indicated derivatives were normalized to an OD_600_ of 0.5, serially diluted 10-fold, and spotted (5 μl) onto agar medium with or without 0.4 μg/ml EMB as indicated. Plates were incubated for 36 hours at 30°C and photographed. Note that a lower concentration of EMB was used in this experiment relative to that shown in Fig 3C in order to better discern the severity of the EMB hypersensitivity phenotype for each strain.

In addition to RipC, *Cglu* encodes a second PG hydrolase called RipA that plays a role in cell separation [27]. Unlike a Δ*ripC* mutant, cells inactivated for RipA alone were not found to display a separation defect [27]. However, deletion of *ripA* in a Δ*ripC* background was found to exacerbate the cell separation defect displayed by cells in which only RipC was inactivated [27]. We re-created these mutants in the MB001 background and confirmed these findings (**S6 Fig**). Furthermore, we found that although deletion of *ripA* alone did not change the EMB-sensitivity of MB001 cells, it enhanced the EMB sensitivity of Δ*ripC* cells (**Fig 5**). These results suggest that RipA and RipC contribute to distinct cell separation pathways. To determine whether SteA and SteB contribute to one of these pathways or form yet another mode of cell separation, we combined the *ste* deletions with those of *ripC* or *ripA* and assessed their EMB sensitivity. Deletion of *ripA* was found to enhance the EMB sensitivity of cells deleted for *steA* or *steB*, but Δ*steA* Δ*ripC* and Δ*steB* Δ*ripC* mutants displayed the same EMB sensitivity as the single *ste* deletion mutants (**Fig 5**). We therefore conclude that SteA and SteB are likely functioning as part of the RipC cell separation pathway. Further support for this possibility is presented below.

### Mutants defective for SteA, SteB, RipC, and FtsEX are delayed for V-snapping

The multiple septa observed in the Δ*steA*, Δ*steB*, Δ*ripC*, *and* Δ*ftsEX* mutants could either be due to a defect in cell separation or to septal closure being slowed such that new ones form before the old septa are completed. Like other bacteria, cell division in *Cglu* is mediated by a cytokinetic ring apparatus organized and controlled by polymers of the tubulin-like protein FtsZ [33]. We monitored the lifetime of the Z-ring constriction process as a proxy for the rate of septal closure. A fluorescent monomeric superfolding variant of GFP (msfGFP) fused to FtsZ (msfGFP-FtsZ) was produced from an ectopic site on the chromosome in the wild-type and mutant strains, and the lifetime of the Z-ring from formation to resolution was monitored by time-lapse microscopy. In the same cells, the progress of septum completion was monitored with a fluorescent trehalose reporter, 6-TMR-Tre [34]. This labeled molecule is incorporated into the non-covalently attached trehalose glycolipids that form the outer leaflet of the mycomembrane. Previous work has shown that 6-TMR-Tre infiltrates the newly completed septum through perforations in the peripheral peptidoglycan just before the daughter cells abruptly separate via a mechanical fracturing event called V-snapping [26]. The time delay between 6-TMR-Tre infiltration and V-snapping therefore provides a useful kinetic parameter with which to characterize the efficiency of the cell separation process.

**Fig 6:**
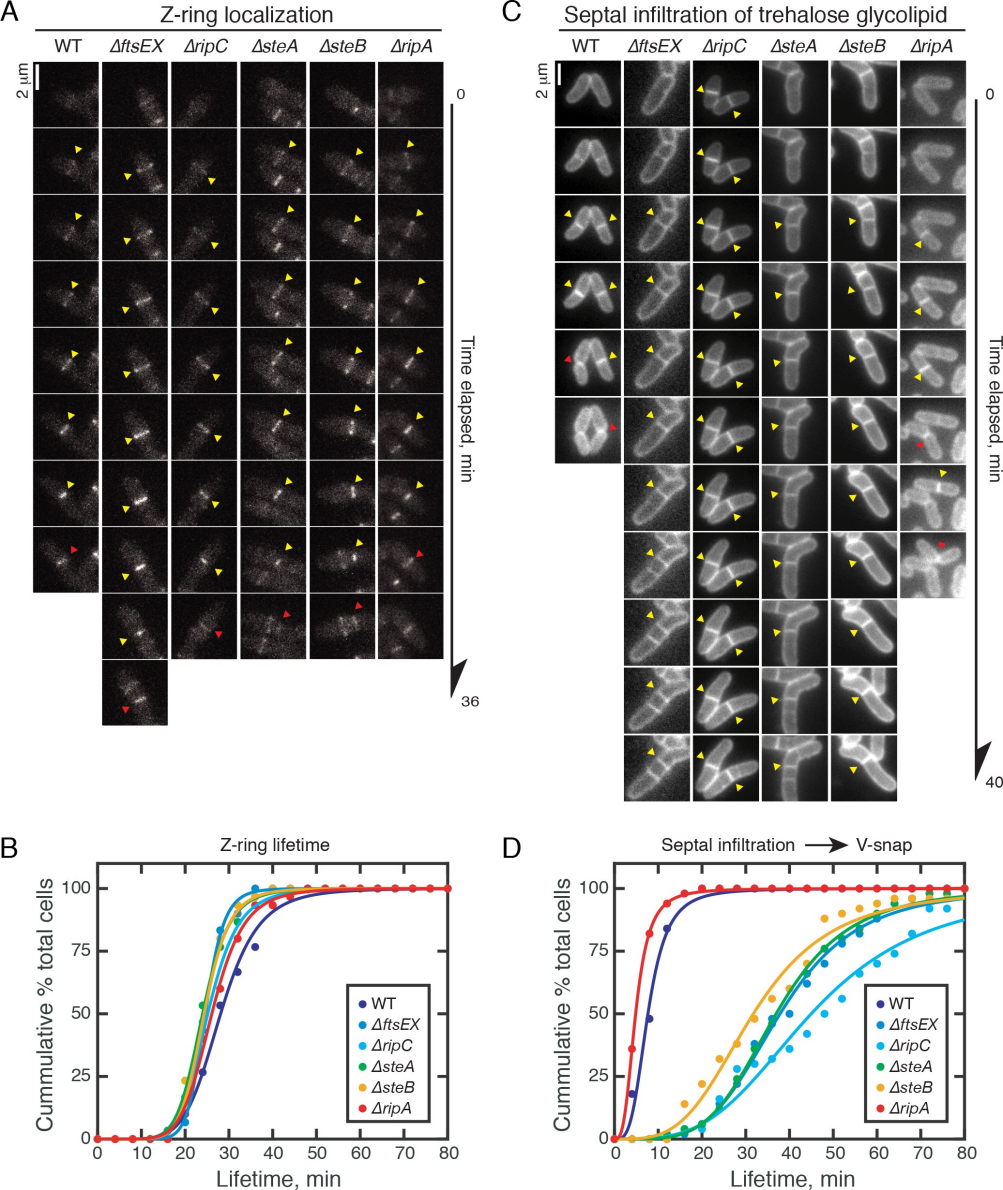
Assessment of the cell division phenotype of *ste* mutants. (**A**) Shown are representative time-lapse fluorescence images used to follow Z-ring dynamics in MB001(WT) cells and its indicated derivatives. Yellow arrowheads denote Z-ring formation, and they transition to a red arrowhead at the time of observed resolution (note the expansion of the fluorescent band). These strains expressed *msfGFP-ftsZ* from the integrated pHCL106 plasmid as well as native *ftsZ*. Overnight cultures were diluted 1:1000 in BHI and grown at 30°C. When the OD_600_ reached 0.1, cells were loaded into a CellAsic microfluic plate and imaged by phase-contrast and fluorescence microscopy at 4-minute intervals. (**B**) Quantification of the Z-ring lifetime for each strain. The life-time of the Z-ring was defined as the time elapsed between Z-ring formation and its resolution, which was observed as the transition from a constricted band to an expanded band of lower fluorescence intensity. Z-ring lifetimes were manually measured for each strain and plotted as the cumulative percentage of cells with a lifetime less than or equal to a given time on the X-axis. The lifetime of thirty Z-rings was quantified for each strain. (**C**) The same cells in (A) were also monitored for the infiltration of trehalose glycolipid by exposing the cells with BHI supplemented with 100 μM 6-TMR-Tre for two minutes every 1 hour during imaging [34]. Representatives time lapse fluorescence images taken at 4 min intervals are shown for each strain. Yellow arrowheads denote septal infiltration of trehalose glycolipid and red arrows denote the V-snapping event. Note that the septa of *ΔftsEX*, *ΔripC*, *ΔsteA* and *ΔsteB* mutants eventually undergo V-snapping, but they are not shown here due the extreme delay. (**D**) V-snap delay was defined as the time elapsed between infiltration of trehalose glycolipid at the septum and V-snapping. It was quantified manually for each strain shown in (**C**) and plotted as cumulative percentage of cells with a V-snap delay less than or equal to a given time on the X-axis. Fifty infiltration events were quantified for each strain. For (**C**) and (**D**), solid lines represent best-fit curves of each dataset with the Hill equation: cumulative percentage = $\frac{100t^{n}}{\tau^{n}+t^{n}}$, in which τ characterizes the lifetime of Z-ring or the V-snap delay.

In wild-type cells and all of the mutants assayed, the characteristic lifetime of the Z-ring constriction process remained unchanged (**Fig 6A and 6B**). However, while wild-type and the Δ*ripA* mutant showed the expected short lag between septal infiltration of trehalose glycolipid and V-snapping (7.6 and 4.8 min, respectively), the time between these two events was significantly extended in the Δ*steA*, Δ*steB*, Δ*ripC*, *and* Δ*ftsEX* mutants (between 31.7 and 45 min) (**Fig 6C and 6D**). We therefore conclude that the cell division defect in these mutants is the same and related to the cell separation process, not slowed septal closure.

### SteA and SteB are recruited to the division site

**Fig 7:**
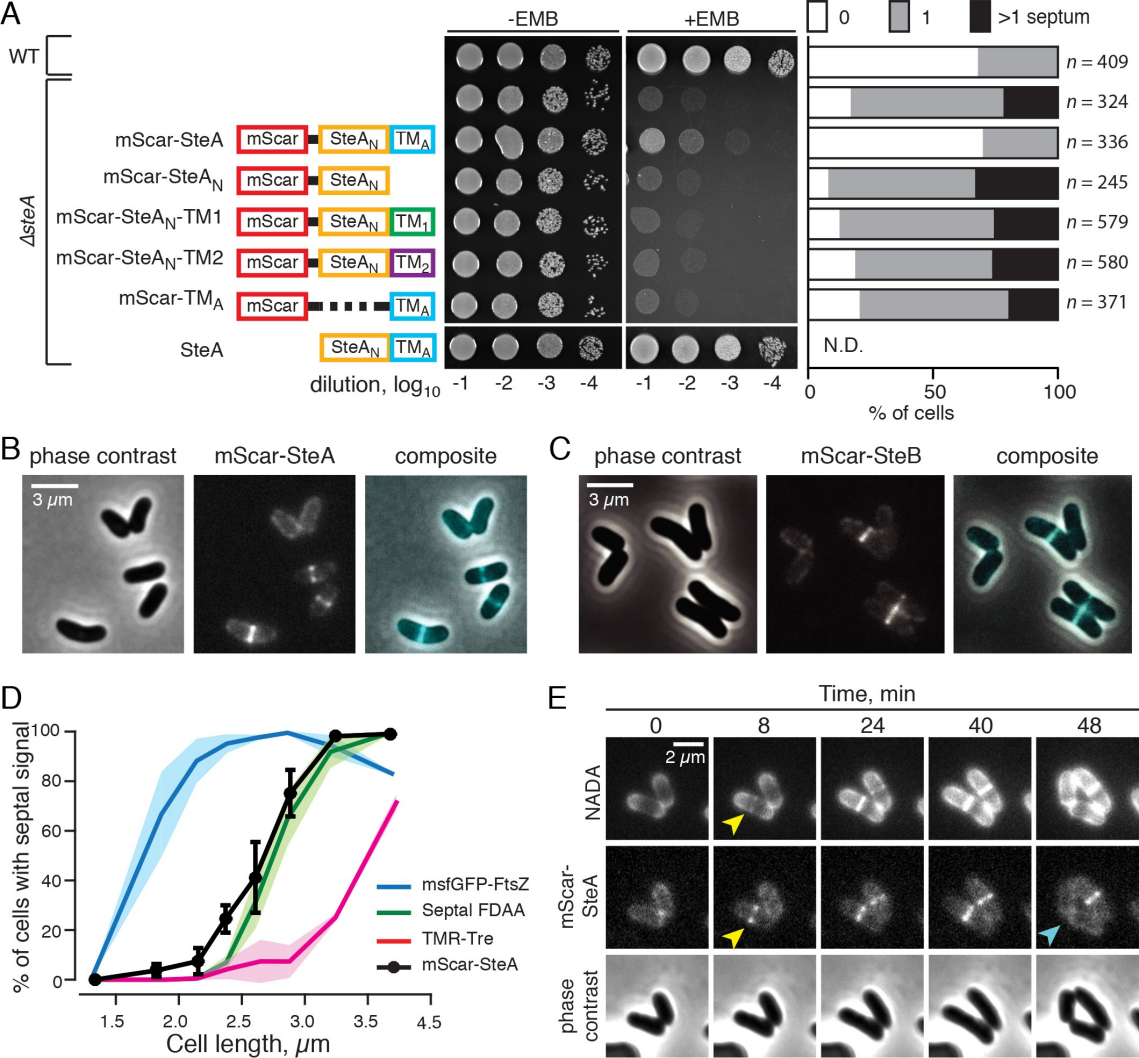
SteA and SteB localize to the division site. (**A**) Functionality of SteA variants. Constructs encoding the indicated mScar-SteA variants under P_*steA*_ control were integrated in the genome of the *ΔsteA* mutant. To quantify the number of septa per cell, the indicated strains were grown in BHI that was supplemented with NADA to label PG for five hours before imaging by phase-contrast, and for fluorescence (green and red channels). SteA_N_ indicates the N-terminal portion of SteA covering residue 3–347. TM_A_ indicates the C-terminal portion of SteA (residue 331–393) containing the transmembrane domain. Constructs encoding the indicated SteA variants are as follows: pHCL171: mScar-SteA; pHCL172: mScar-SteA_N_; pHCL173: mScar-SteA_N_-TM_1_; pHCL174: mScar-SteA_N_-TM_2_; pHCL175: TM_A_; and pHCL57: SteA. Septa per cells were scored using the Cell Counter plugin in the FIJI software [59] and the percentage of cells with zero, one or more than one septum were calculated and plotted. (**B-C**) Phase-contrast and fluorescence micrographs of *Cglu* cells producing mScar-SteA (**B**) or mScar-SteB (**C**), as the sole copy of the corresponding gene, from integrated plasmids pHCL171 or pHCL170, respectively. (**D**) Correlation of cell length-dependent mid-cell accumulation of mScar-SteA with septal PG synthesis, the infiltration of the trehalose glycolipid, and Z-ring formation at the population level. Overnight cultures of the appropriate strains were diluted 1:1000 in BHI and grown at 30°C. When the OD_600_ reached 0.2–0.3, cells were processed (when necessary) and spotted on an agarose pad for phase-contrast and fluorescence microscopy. Cells were grouped according to their cell lengths into bins of 0.3 μm increments and the percentage of cells with septal signal were calculated for each bin. With the exception of mScar-SteA (see below), solid lines represent the mean value while the shaded areas represent standard error of the mean. To follow the timing of Z-ring assembly, exponentially growing MB001(*att*B1HCL106) cells expressig msfGFP-FtsZ were used (*n =* 2, 535 cells and 734 cells, respectively). To visualize mid-cell localization of SteA, HL2(*att*B1HCL171) cells were used (*n =* 157 and 879 cells, respectively). Black filled spheres represent mean values and error bars represent standard error of the mean. Septal PG labeling and trehalose-glycolipid infiltration were followed in both strains. For septal PG labeling, exponentially growing cells were pulse-labeled with 1 μM NADA for 5 min before imaging. The plotted curve represents the average of two highly similar curves (low standard error) obtained from the two strains MB001(*att*B1HCL106) (*n* = 734) and HL2(*att*B1HCL171) (*n* = 879). For trehalose-glycolipid labeling, cells were grown in the BHI medium supplemented with 100 μM 6-TMR-Tre for 5 min and washed twice with BHI by gentle pelleting and resuspension before imaging. As with the septal PG labelling, the curve represents the average of the results from the two strains: MB001(*att*B1HCL106) (n = 535) and HL2(*att*B1HCL171) (n = 330). (**E**) Time-lapse microscopy showing coincidental mid-cell accumulation of mScar-SteA and commencement of septal PG construction. Unlabeled HL2(*att*B1HCL171) cells in the exponential growth phase were loaded in a CELLASIC ONIX microfluidic device. Five minutes before the start of time-lapse microscopy, the cells were exposed to medium supplemented with 1 μM NADA to label sites of active peptidoglycan synthesis (see Methods for details). Images were taken at 8 minutes intervals. Yellow arrows indicate concomitant appearance of NADA and mScar-SteA signals at the cell septum. Cyan arrow indicates dissolution of the septal mScar-SteA signal upon V-snapping.

To investigate whether SteA and SteB are likely to be playing a direct role in the cell separation process, we assessed their subcellular localization. Fusions with mScarlet-I (mScar) [35] were constructed and produced from the *att*B1 site in the chromosome. Both mScar-SteA and mScar-SteB fusions restored the normal cell length distribution to cells lacking the native protein (**S7A–S7C Fig**). The mScar-SteA fusion was also found to reduce the number of observed septa per cell back to normal (**Fig 7A**). However, there was a notable difference in the ability of the fusions to rescue the EMB-sensitivity of mutant cells. The mScar-SteB fusion came close to fully complementing the EMB^S^ phenotype of *ΔsteB* mutants (**S7D Fig**). By contrast, mScar-SteA only weakly rescued the EMB-sensitivity of *ΔsteA* cells (**Fig 7A**), indicating that there is not a perfect correspondence between cell separation and EMB resistance. We infer from this result that SteA may have a second function unrelated to cell separation and that the fusion may be defective for this activity (See Discussion).

Cells expressing mScar-SteA or mScar-SteB fusions displayed one of two localization patterns. In recently separated cells that remained tethered to their siblings, the mScar signal decorated the cell periphery, consistent with a membrane localization (**Fig 7B and 7C**). In cells that were presumably further along in the division cycle, cells displayed tight band of fluorescence at the prospective division site with minimal signal observed along the cell periphery (**Fig 7B and 7C**). Thus, SteA and SteB are recruited to the cytokinetic ring and are likely to be directly participating in the division process.

We next investigated the timing of SteA recruitment to the division site relative to other measurable cell division events: Z-ring formation assessed using msfGFP-FtsZ, septal PG construction assessed by labeling of the septum with the FDAA called NADA [32], and the infiltration of labeled trehalose-glycolipids into cell septum. The analysis was carried out using population level measurements of individually labeled cells to determine the cell length (a proxy for age) at which 50% of the population displayed label localized at the division site. As expected, Z-ring formation, septal PG synthesis, and infiltration of trehalose-glycolipids at the cell septum occurred in apparent chronological order with characteristic cell lengths of septal labeling of 1.76, 2.75, and 3.49 μm, respectively (**Fig 7D**). The characteristic cell length of mScar-SteA recruitment to the division site was 2.64 μm, which coincides with the inception of septal PG assembly (**Fig 7D**). To further investigate the relative timing of these two events, we simultaneously monitored nascent PG synthesis and mScar-SteA localization at the single-cell level in a time-lapsed experiment. Cells producing mScar-SteA were imaged within a microfluidic device, which allowed constant infusion with media containing NADA. Consistent with the population level analysis, in every one of the more than thirty cells that were manually monitored in time-lapse, the mScar-SteA and NADA signals appeared at the division site simultaneously (**Fig 7E**). Furthermore, the mScar-SteA signal was observed to disperse from the cell septum immediately following V-snapping (**Fig 7E**), indicating that SteA does not linger at the new cell poles following division.

### Septal localization of SteA requires its cytoplasmic and transmembrane domains

Next, we investigated the domain requirements for SteA localization and function. A series of mScar-SteA variants were constructed in which different parts of the protein were either deleted or substituted with another domain (**Fig 7A**). Even though all of the protein fusions accumulated to comparable levels in *Cglu* (**S8 Fig**), only the full-length mScar-SteA protein was capable of rescuing the the cell separation phenotype of the *ΔsteA* mutant or partially rescuing EMB hypersensitivity (**Fig 7A**). In accordance with the functionality of the fusions, only mScar-SteA showed specific enrichment at the septum (**S9 Fig**). The N-terminal domain of SteA (mScar-SteA_N_) displayed diffuse cytoplasmic signal, whereas mScar fused to the C-terminal transmembrane domain of SteA (mScar-TM_A_) labeled the peripheral membrane as well as septal membranes (**S9 Fig**). To test whether the native SteA transmembrane domain plays a role in SteA function or localization beyond anchoring the N-terminal domain to the membrane, it was replaced by unrelated transmembrane domains from two putative tail-anchored membrane proteins of *Streptomycetes coelicolor*: SecE (TM_1_) and PkaB (TM_2_) [36]. These constructs, mScar-SteA_N_-TM_1_ and mScar-SteA_N_-TM_2,_ displayed a peripheral localization signal consistent with membrane recruitment, but they were not functional and did not promote specific septal labeling (**Figs 7A and S9**). We conclude that septal localization of SteA is critical for proper cell separation and that both the N-terminal and transmembrane domains of the proteins are required for its function.

### SteB promotes robust septal localization of SteA

**Fig 8:**
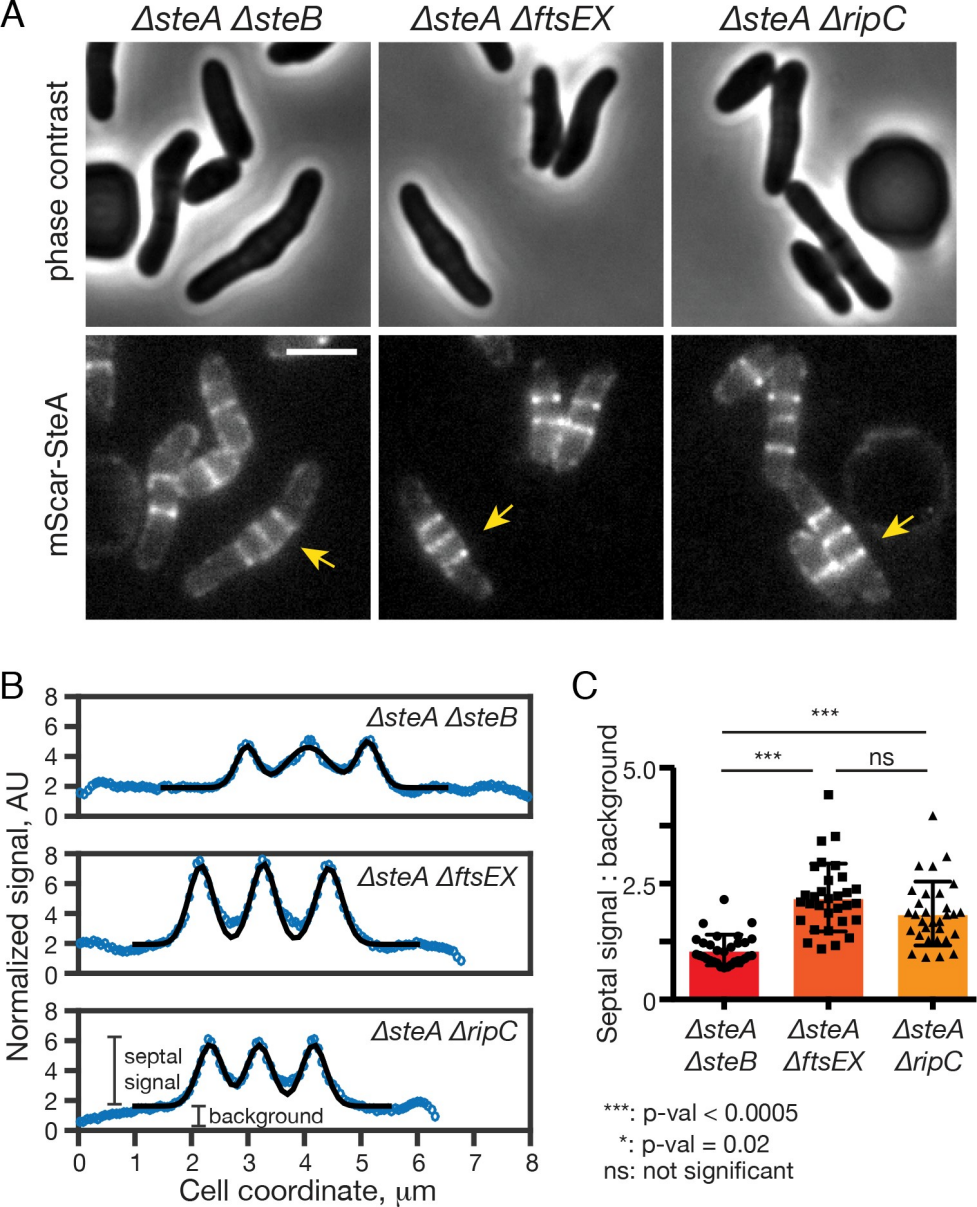
SteB is required for robust recruitment of SteA to the division site. (**A**) Representative images of the indicated *Cglu* mutants producing mScar-SteA under P_*steA*_ from genome integrated pHCL171. Overnight cell cultures were diluted 1:1000 in BHI and grown at 30°C. When the OD_600_ reached 0.2–0.3, cell cultures were diluted 10-fold and loaded in a CELLASIC ONIX microfluidic device for phase-contrast and fluorescence microscopy. Scale bar: 2 μm. (**B**) Reduced accumulation of mScar-SteA signal at the division site in cells lacking SteB. Representative examples of mScar-SteA fluorescence profiles in the indicated strains using cells in (**A**) marked by yellow arrows. Cell detection and fluorescence quantification was performed using Oufti [60]. To quantify the background signal and the septal signal, the fluorescence profiles were fitted with multi-exponential Gaussians plus background (black line) using Matlab. The height of each Gaussian peak was taken as the value of septal signals. (**C**) Bar graphs comparing the septal mScar-SteA signal to background ratio of the indicated strains (31 septa from at least 18 cells from each strain were used for measurements). P-values (Tukey-Kramer test) report the statistical significance between the difference of the means using Prism software.

To determine whether any of the other factors required for cell separation are required for the recruitment of SteA to the division site, we visualized the localization of mScar-SteA in mutants inactivated for SteB, RipC, or FtsEX. All of these mutant strains contain multiple septa resulting from their cell separation defect. In each case mScar-SteA labeled these septa (**Fig 8A**). However, we noticed that the intensity of mScar-SteA septal bands in the *ΔsteB* mutant was consistently lower when compared to the other two mutants. The lower septal mScar-SteA signal in *ΔsteB* cells was not due to a reduction in the overall mScar-SteA protein level (**S10 Fig**). Rather, we measured a statistically significant increase in the non-septal mScar-SteA signal in the *ΔsteB* mutant (2.74 ± 0.17, 23 cells) compared to those of *ΔftsEX* (1.88 ± 0.09, 18 cells) or *ΔripC* cells (2.19 ± 0.14, 24 cells) (**Fig 8B**). As a result, the ratio of septal mScar-SteA signal to background in the *ΔsteB* mutant (1.07 ± 0.37) was significantly reduced compared to mutants inactivated for *ftsEX* (2.20 ± 0.72) or *ripC* (1.85 ± 0.72) (**Fig 8C**). These results indicate that SteB is required for optimal recruitment of SteA to the division site. The observation that SteA retains some ability to localize in the absence of SteB may explain why the *ΔsteB* mutant exhibits a milder EMB sensitivity phenotype than cells lacking SteA.

### SteA and SteB form a complex that interacts with RipC

**Fig 9:**
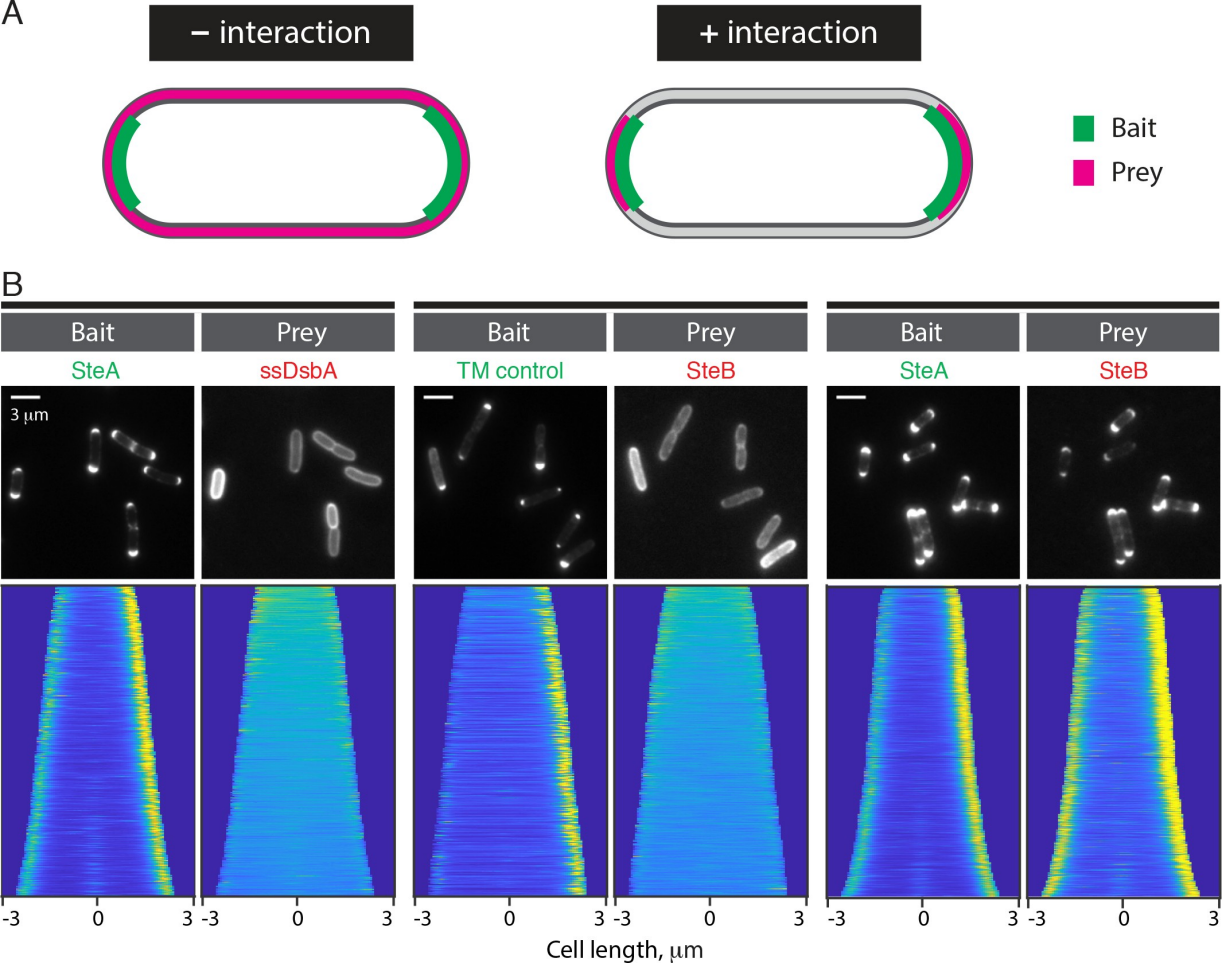
SteA and SteB form a complex. (**A**) Schematic depicting the expected localization patterns of a prey depending on whether or not it interacts with the bait in the POLAR two hybrid assay. (**B**) *Top row*: representative fluorescence images of TB28 *E*. *coli* cells expressing the indicated the bait and prey proteins. The indicated baits were expressed using the following medium-copy plasmids: pHCL149 (TM control) and pHCL202 (SteA). The TM control harbors residues 2–55 of *Pseudomonas aeruginosa* PAO1 PonB covering its transmembrane domain that has a N-in/C-out topology. The preys were expressed from genome-integrated plasmids (pHCL147: ^ss^DsbA and pHCL194: SteB). Cell cultures were grown from a single colony in LB supplemented with antibiotics at 37°C for two hours. To induce the bait and prey proteins, cells were collected by centrifugation and resuspended in M9 supplemented with 0.2% arabinose and 100 uM IPTG and grown for two hours at 37°C. Cells were spotted on an agarose pad for imaging. *Bottom row*: demographs showing fluorescence distribution of the corresponding prey and bait fusions at the population level. Single-cell fluorescence quantification was performed using Oufti [60]. Cells were oriented using a custom-written MATLAB script such that the cell pole with the higher bait fluorescence was located on the right of the demograph. At least 250 cells were used to generate each demograph.

The requirement of SteB for robust recruitment of SteA to the division site suggested that the two proteins might interact at the septum. To investigate their potential interaction further, we employed a two hybrid assay we recently developed called POLAR based on PopZ-linked apical recruitment in *E*. *coli*. This method takes advantage of the polar organizing protein called PopZ from *Caulobacter cresentus* [37–39] and its ability to form polar foci in *E*. *coli*. Bait proteins are fused to the H3H4 peptide of PopZ and sfGFP. When they are produced in *E*. *coli* cells that also make full-length PopZ, the bait fusions are recruited to the cell pole by the H3H4 peptide-PopZ interaction (**Fig 9A**). Prey proteins are fused to mScar and their interaction with the bait is assessed based on whether or not they are also recruited to the pole (**Fig 9A**). The assay is thus similar to previous cytological interaction detection systems based on DivIVA fusions [40,41], but it benefits form the more robust and specific polar accumulation of PopZ in *E*. *coli* relative to DivIVA. An in-depth description of the POLAR method and the types of protein-protein interactions it can detect will be presented in a separate report.

To assess the SteA-SteB interaction, H3H4-sfGFP-SteA was used as the bait. The fusion successfully resulted in the recruitment of SteB-mScar to the cell pole whereas a control membrane-anchored bait protein H3H4-sfGFP-TM did not (**Fig 9B**). Additionally, a control periplasmic mScar protein was also not recruited to the pole by H3H4-sfGFP-SteA (**Fig 9B**). In addition to visual inspection of individual cells, the POLAR results can also be viewed at a population level using a demograph in which quantified fluorescence traces of cells are stacked according to cell length and visualized as a heat map. Such population level results also support a specific interaction between the H3H4-sfGFP-SteA bait and SteB-mScar (**Fig 9B**). Given that *E*. *coli* is only distantly related to *Cglu* and unlikely to encode interaction partners for *Cglu* proteins, we conclude from the POLAR results that SteA and SteB are likely to interact directly to form a complex.

**Fig 10:**
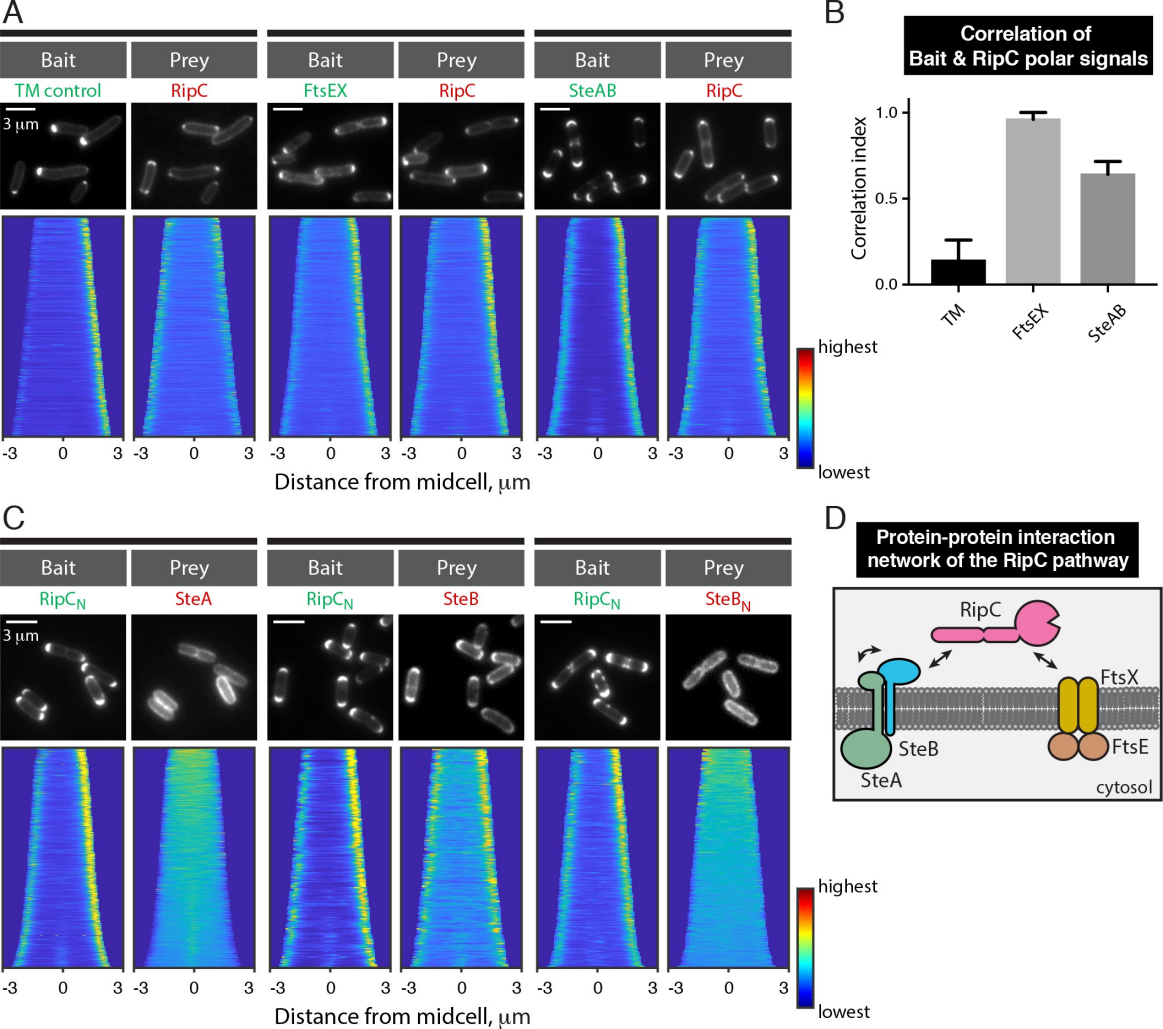
RipC interacts with both the SteAB and FtsEX complexes. (**A** & **C**) *Top row*: representative fluorescence images of TB28 *E*. *coli* cells expressing the indicated the bait and prey proteins. The indicated baits were expressed using the following plasmids: pHCL149 (TM control), pHCL204 (SteAB), pHCL205 (FtsEX), pHCL213 (RipC_N_). The preys were expressed from genome-integrated plasmids (pHCL221: RipC, pHCL214: SteA, pHCL194: SteB and pHCL196: SteB_N_). Cells were grown and imaged as in **Fig 9B.** (**B**) Fluorescence correlation between the bait and the prey at the cell pole. To quantify the relative distribution of the the bait and prey between the poles, we first calculated fractional difference of the bait signal (*D*_*bait*_) and the prey signal (*D*_*prey*_) between the poles. The correlation between the bait and the prey signal distribution was represented by the correlation index (*CI*), which is the ratio of *D*_*prey*_ to *D*_*bait*_. *CI* value of 1 indicates complete positive correlation, 0 indicates no correlation while -1 indicates complete negative correlation. A least 215 cells were used in each calculation (*n* = 2 for the FtsEX experiment and *n* = 3 for the control and the SteAB experiments). (**D**) Summary of the protein-protein interaction network of the RipC cell separation pathway.

We next wanted to assess the ability of the SteAB complex to interact with RipC. However, the assay was complicated by the RipC-mScar localization pattern in cells producing the control bait fusion H3H4-msfGFP-TM, which it is not expected to interact with. The bait displayed a primarily unipolar localization signal as expected, but RipC-mScar localized to both poles in a manner uncorrelated to the pattern of the control bait (**Fig 10A and 10B**). Even though cells in the demograph were oriented such that the pole with higher bait accumulation was oriented to the right, RipC-mScar intensity was distributed roughly equally between the two poles (**Fig 10A**). This bipolar localization of RipC-mScar in the *E*. *coli* periplasm likely reflects some level of aggregation due to the predicted intrinsically disordered and coiled coil domains present in the N-terminal portion of RipC. Accordingly, a RipC truncation mutant lacking the C-terminal NlpC/p60 catalytic domain (^ss^DsbA-mScar-RipC_N_) also showed bipolar localization when it was secreted to the *E*. *coli* periplasm (**S11A Fig**). Nevertheless, when H3H4-msfGFP-FtsEX was used as the bait, the mScar-RipC signal became biased to the pole with the highest bait signal. In this case the correlation index (CI) between the FtsEX bait and the RipC prey localization pattern was 0.96 ± 0.04, much higher that the 0.14 ± 0.12 index value between RipC and the control bait (**Fig 10B**). Given that FtsEX and RipC are known to interact robustly in mycobacteria, we conclude that the POLAR assay can accurately detect RipC interactions despite its tendency to generate a bipolar signal when fused to mScar. We therefore assessed whether or not RipC interacts with SteAB using H3H4-msfGFP-SteA as a bait co-expressed with untagged SteB. As with FtsEX, the intensity of the polar RipC-mScar signal was highly correlated with the H3H4-msfGFP-SteA bait signal (CI = 0.64 ± 0.07), albeit to a slightly lesser degree than FtsEX (**Fig 10B**). A similar correlation of polar signals (CI = 0.67 ± 0.12) was obtained when a prey harboring only the N-terminal domain of RipC was used instead of the full-length protein (**S11A and S11B Fig**). This result indicates that RipC interacts with the SteAB complex primarily if not entirely through its N-terminal domain. To determine which component(s) of the SteAB complex directly contacts RipC, we used the N-terminal domain of RipC fused to H3H4-msfGFP via a TM domain (H3H4-msfGFP-TM-RipC_N_) as the bait. It recruited SteB-mScar but not mScar-SteA prey to the cell poles (**Fig 10C**), suggesting that SteB directly binds RipC via its N-terminal coiled-coil rich domain. This interaction requires the C-terminal part of SteB as a SteB variant lacking the extracellular domain (SteB_N_-mScar) failed to be recruited to the cell pole by the RipC_N_ bait (**Fig 10C**). Notably, SteB_N_-mScar retains its ability to interact with SteA (**S11C Fig**). Using the POLAR assay, we were unable to detect a direct interaction between FtsEX and SteA or SteB (**S11D Fig**). Overall, the interaction data suggest that SteAB forms part of the FtsEX-RipC complex at the septum in *Cglu* to promote septal PG remodeling and daughter cell separation via V-snapping (**Fig 10D**).

## Discussion

A better understanding of cell envelope biogenesis in the Corynebacterineae will enable the development of novel treatments for diseases caused by *Mtb*, nontuberculosis mycobacteria, and pathogenic corynebacteria. Over the years, *Cglu* has been a useful model for elucidating essential steps in the assembly of the complex and multilayered envelope unique to this class of bacteria. To enhance our ability to use *Cglu* as a system for discovering fundamental mechanisms required for proper envelope assembly in the mycolata, we generated the first high-density library of transposon mutants in *Cglu* and performed a global genetic analysis of gene function in this organism using Tn-Seq. By defining the essential gene content of *Cglu* for comparison with similar datasets available for *Mtb* [18,42,43], the results provide an important resource for researchers studying the growth of this group of organisms. To focus the analysis on envelope assembly, we also used Tn-Seq to identify loci that when inactivated result in hypersensitivity to the AG biogenesis inhibitor EMB. The utility of the datasets generated was demonstrated through the identification of new and conserved components of the division machinery that are required for proper daughter cell separation.

Like other members of the Corynebacterineae, *Cglu* cells complete cell division by rapidly splitting at their division septum to form two daughters [44]. The process is aptly referred to as V-snapping, and was recently characterized in detail using time-lapse microscopy and fluorescent reporters to label different layers of the envelope [26]. It was found that the envelope layers were assembled sequentially at the division site. The PG layer was the first component of the septum to be observed, followed shortly afterwards by reporters of covalently linked mycolic acids, which presumably report on both the AG layer and its attached lipids being assembled at the division site. Finally, free labeled trehalose glycolipids were observed to infiltrate the septum from the surrounding mycomembrane, and V-snapping separation took place shortly afterwards. Diffusion of trehelose glycolipids into the septum was associated with the visualization of perforations in the peripheral PG layer. What forms these initial imperfections in the PG is not clear, but their enlargement appears to require the PG hydrolase RipC [26]. Ultimately, these perforations are thought to elicit the mechanical fracture of the septum and the rapid V-snapping event [26,44].

The mechanisms that control the activity of cell separation hydrolases like RipC have been investigated in diverse organisms. In many bacteria, a key role for the FtsEX ABC-transporter like complex has been uncovered. In *E*. *coli* and *S*. *pneumoniae*, FtsEX associates with EnvC and PcsB, respectively [28,29]. Both partner proteins have an N-terminal coiled-coil domain that associates with an external loop domain of FtsX and a C-terminal domain with homology to PG hydrolases [28,29]. This effector domain of PcsB is thought to directly cleave PG whereas for EnvC it has been shown to activate cell wall cleavage by amidases [45]. In both cases, the proteins localize to the division site to promote daughter cell separation, and this function requires the ATPase activity of FtsE [28,29]. It has therefore been proposed that FtsEX promotes cell wall remodeling using ATP-driven conformational changes in the complex to activate PG hydrolysis by its partner protein. A similar role for FtsEX in cell division has since been uncovered in *C*. *cresentus* [46]. Additionally, FtsEX has been implicated in controlling cell wall cleavage by CwlO in *B*. *subtilis* [47], but in this case it is required to promote cell wall expansion during cell elongation rather than cleavage of the cell wall septum.

In the Corynebacterineae, the external loop domain of *Mtb* FtsX has been shown to interact with RipC *in vitro* and to modestly enhance its PG cleavage activity [30]. RipC has also been shown to be recruited to the division site in *Cglu* and inactivation of RipC or FtsEX has been associated with a cell separation defect [27,31]. Here, we have identified SteA and SteB as new components of the cytokinetic ring that promote the V-snapping process. Phenotypically, mutants lacking SteA or SteB are largely indistinguishable from those inactivated for RipC or the FtsEX complex. Interaction studies using the POLAR assay indicate that SteA and SteB are likely to form a complex and that the external domain of the SteB component is also likely to interact with the N-terminal domain of RipC. Our interaction results also suggest that RipC and the FtsEX complex interact in *Cglu*. It is interesting to note that the N-terminal domain of RipC is significantly longer that the corresponding domains of EnvC and PcsB (**S12 Fig**), suggesting that this extension might allow for the simultaneous interaction of RipC with SteB and FtsEX. Based on all of the observed interactions, we propose that a multiprotein complex of SteAB-RipC-FtsEX forms at the division site to promote cell wall cleavage and generate the septal imperfections that eventually lead to its mechanical fracture and V-snapping.

In *E*. *coli*, FtsEX has been shown to interact with the FtsA protein, a highly conserved component of the divisome that functions in part to anchor filaments of FtsZ to the membrane [48]. The FtsEX-FtsA interaction has been implicated in the activation of PG synthesis by the cytokinetic ring [49]. Therefore, the FtsEX complex in *E*. *coli* is thought to link the processes of cell wall synthesis and remodeling at the division site [49]. Notably, Corynebacterineae lack FtsA, but almost universally encode SteAB. Thus, although the precise function of the SteAB complex in cell separation remains to be determined, an attractive possibility is that SteAB serves as an alternative connector that links RipC-FtsEX with other functions of the division machinery. A clue to this possible connection comes from the observation that an mScar-SteA fusion corrected the cell separation defect of a Δ*steA* mutant but not its hypersensitivity to the AG synthesis inhibitor EMB. Thus, rather than PG synthesis, SteAB may link RipC-FtsEX activity with AG biogenesis. Intriguingly, a complex of two small membrane proteins (SweC-SweD) unrelated to SteAB was recently found to be required for the cell wall expansion activity of the FtsEX-CwlO complex in *B*. *subtilis* [50]. Being involved in cell elongation as opposed to cell division, this PG remodeling system is also unlikely to interface with FtsA. Thus, the use of accessory membrane protein partners to modulate the function of FtsEX complexes may be a common feature employed by bacteria when the system is not connected with FtsA in the context of the division machinery.

Overall, our results with SteAB highlight the potential of employing global genetic approaches in *Cglu* to provide insight into the mechanisms of mycolata envelope assembly. Continued mining of this dataset as well as additional Tn-Seq screens will likely lead to a wealth of new discoveries that should help us learn more about how members of the Corynebacterineae grow and how we can best interfere with the process for antibiotic development.

## Methods

### Media, bacterial strains, and plasmids

All *Cglu* strains used are derivatives of MB001 [14]. Unless mentioned otherwise, strains were grown in Brain-Heart Infusion Medium (BHI) that was supplemented with 15 μg/mL kanamycin (Kan), when necessary. Plasmids were maintained in either DH5a(λpir) or S17-1(λpir). All *E*. *coli* strains used in the reported POLAR two-hybrid experiments are derivatives of TB28 [51] and are grown in LB (1% tryptone, 0.5% yeast extract, 0.5% NaCl). Whenever necessary, antibiotics were used at 25 (Kan), 15 (chloramphenicol; Cm), 50 (ampicillin; Amp) or 5 (tetracycline; Tet) μg/mL. Growth conditions for microscopy experiments are described in the figure legends and the appropriate Methods section. Detailed information about plasmid constructions can be found in S1 Text. All strains are listed in S4 Table and all plasmids are listed in S5 Table.

### Gene deletion in *Cglu*

We used the temperature-sensitive plasmid pCRD206 to perform allelic replacement in *Cglu* essential as described previously [52] with some modifications. Briefly, sequences corresponding to approximately 700 bp upstream and downstream of the desired deletion were inserted into pCRD206. The resulting plasmid was transformed into the appropriate recipient strain. Transformants were selected and propagated on BHI-Kan agarose plates at 25°C. A few colonies were then purified on BHI-Kan agarose and grown at 37°C for 36 hours. One or two of the resulting Kan^R^ colonies were then grown in BHI liquid medium lacking antibiotic at 25°C overnight. The lower temperature allows replication from the plasmid origin, which selects for a second recombination to remove the plasmid from the chromosome. An aliquot of the overnight culture (10 μL) was then spread on a BHI agarose plate supplemented with 10% sucrose, which further selects against *sacB* in the vector, and the plate was then incubated overnight at 30°C. The resulting colonies were replica patched onto BHI and BHI-Kan plates to identify Kan^S^ colonies lacking plasmid. Deletion alleles in Kan^S^ isolates were finally confirmed by colony PCR.

### Plasmid integration at the *att*B1 site in *Cglu*

For complementation studies, genes were integrated at the *att*B1 site on the chromosome using the pK-PIM vector [53]. The plasmid was introduced into the recipient by conjugation.

### Conjugation into *Cglu*

Plasmids were transferred into *Cglu* using the *E*. *coli* strain S17-1(λpir). Conjugation was performed according to [54] with some modifications. Briefly, overnight cultures of recipient strains grown in LB supplemented with 4% (w/v) glucose (LB-Glu4) were diluted 1:5 in the same medium and incubated for two hours at 30°C. After adjusting the density to an OD_600_ of 2.0, each culture was incubated in a 48.5°C water bath for 9 minutes. Overnight cultures of the donor strain were diluted 1:100 in LB supplemented with the appropriate antibiotics and grown at 37°C until OD_600_ reached 1.0. When the recipient cultures cooled to room temperature, the donor and recipient cultures were mixed at 1:3 ratio, gently pelleted to collect cells, which were then resuspended into 150–200 μL LB-Glu4 medium. The cell suspension was pipetted onto a 0.4 μm sterile filter placed on a LB-Glu4 agarose plate and incubated. Following overnight incubation at 30°C, cells were collected from the filter and resuspended into LB and plated on BHI-Kan agar plates that were supplemented with 30 μg/mL nalidixic acid to select against *E*. *coli* donors. Plasmid integration was confirmed by colony PCR using a primer mixture consisting of ATTB1g-UP, ATTB1g-UN, ATTB2g-UP and ATTB2g-UN [53]. Using WT gDNA as the template, the PCR reaction generates two bands of 464 bp and 700 bp in size, which represent uninterrupted *att*B1 and *att*B2 sites respectively. Therefore, specific disappearance of the 464-bp band was used as a diagnostic for plasmid integration at the *att*B1 site.

### Preparation of electrocompetent *Cglu cells*

Cells of *Cglu* were grown to stationary phase, and 10 mL of this culture was diluted into 1 L BHIS (BHI + 91 g/L sorbitol) that was supplemented with 25 g glycine, 0.4 g isoniazid, and 0.1% Tween 80 and incubated shaking at 18°C. The culture was chilled on ice for 1 hour when the OD_600_ reached 0.5 (typically in 16–18 hours). Cells were then pelleted at 4000 x g for 20 min, washed once with 500 ml chilled 10% glycerol, and then three additional times with 100 ml chilled 10% glycerol. The cell density was adjusted to an OD_600_ of 20 before use for electroporation.

### Tn5 transposon insertion library construction and sequencing

The Tn5 transposome was purchased from Epicentre (now discontinued) or prepared in-house using Tn5 transposase purified as described previously [55]. For the in-house preparation, the transposon was amplified by polymerase chain reaction (PCR) using 5’-monophosphorylated primers ME Plus 9–3’ primer (CTGTCTCTTATACACATCTCAACCATCA) and ME Plus 9–5’ primer (CTGTCTCTTATACACATCTCAACCCTGA) using the <KAN-2> transposon supplied by Epicentre as the template. The transposon was purified using the QIAquick PCR purification kit (Qiagen) and eluted with TE buffer (10 mM Tris pH 8.0 and 1 mM EDTA). The Tn5 transposome was reconstituted by vortexing a 1:1 mixture of 1 μM purified transposase [55] with the purified transposon (200 ng/μL) and used immediately after a 30-minute incubation at room temperature. Consistent with another report [56], we found that the Tn5 transposase prepared according to Picelli et al. 2014 loses activity quickly following purification.

To generate mutant libraries, transformation was carried out by electroporating 1 μL of commercial or homemade Tn5 transposome with 100 μl of freshly prepared MB001 electrocompetent cells. Each transformation reaction typically generated between two to five thousand transformants. We pooled transformants from 50 electroporations performed over 4 days to generate our library consisting of approximately 230,000 total transformants.

DNA libraries for Tn-Seq analysis were prepared by a modified version of a published protocol [15]. Genomic DNA was extracted from strains using the Wizard Genomic DNA Purification Kit (Promega) and further purified using Genomic DNA Clean & Concentrator (Zymo). Genomic DNA was fragmented using a Qsonica Q800RS Sonicator for 12 minutes (using a 15 second on and 15 second off pulse cycle) at 20% amplitude. Fragmented DNA was purified with 1.8× volume Agentcourt AMPure XP beads (Beckman Coulter, Inc.) and eluded into 30 μl water. Purified fragmented DNA was then treated with terminal deoxynucleotidyl transferase (TdT; Promega) in a 20 μl reaction with 1 μL 9.5mM dCTP/0.5mM ddCTP, 4 μl 5× TdT reaction buffer and 0.5 μl rTdT at 37°C for 1h, then at 75°C for 20min. TdT-treated DNA was purified with Performa DTR Gel Filtration Cartridge (EdgeBio). Purified, TdT-treated DNA was used as a template in a PCR reaction to amplify the transposon junctions using the Easy-A Hi-Fi Cloning System (Agilent Technologies). The primers used were:

**PolyG-1st-1** 5’-GTGACTGGAGTTCAGACGTGTGCTCTTCCGATCTGGGGGGGGGGGGGGGG-3’ and **Tn5-1st-1** 5’-ACCTGCAGGCATGCAAGCTTCAGGG-3’.

A second nested PCR was then performed to further amplify the transposon junctions and append the sequencing barcode. The primers used were generic NEBNext Multiplex Oligos for Illumina (NEB) and:

**Tn5-2nd-1 5’-**AATGATACGGCGACCACCGAGATCTACACTCTTTTCAGGGTTGAGATGTGTATAAGAGA-3’.

The final product was run on a 2% agarose gel, and fragments ranging from 200–500 bp were gel purified using QIAquick Gel Extraction Kit (Qiagen). Libraries were sequenced at the Tufts University Core Facility on a HiSeq 2500 (Illumina) or a Miseq (Illumina) on a 1× 50 single end run. All sequencing data generated in this study are deposited in Sequence Read Archive under accession PRJNA548135.

Reads were mapped to the *C*. *glutamicum* MB001 genome (NCBI NC_022040.1). To identify EMB sensitive genes, we calculated the fold change in reads between the ethambutol conditions and the no drug conditions. We also performed a Mann-Whitney U test to determine whether differences in the insertion profiles were statistically significant. Genes in which insertions were at least 3-fold enriched or depleted and had a p-value lower than 0.05 by Mann-Whitney were defined as hits. Visual inspection of transposon insertion profiles was performed with the Sanger Artemis Genome Browser and Annotation tool.

### POLAR two hybrid strains construction

Each strain harbors a replicative plasmid that expresses the bait and another genome-integrated plasmid that expresses the prey. We simultaneously introduced, by electroporation, both plasmids into the TB28 recipient that carried the helper plasmid pAH69 [57]. Transformants that integrated the prey plasmid at the HK022 phage attachment site and also harbored the bait plasmid were selected for by plating on LB agarose plates that contained Cm and Tet at 37°C, which prevented the replication of pAH69. Electrocompetent TB28/pAH69 cells were prepared by first diluting an overnight culture into LB supplemented with Amp and incubated shaking at 30°C. When OD_600_ reached 0.15, the culture was shifted to 42°C for 30 minutes to induce integrase expression. The culture was then chilled on ice for 1 hour. Cells were then collected by centrifugation (4000 x g) for 20 min, washed once with 500 ml chilled 10% glycerol, and then three additional times with 100 ml chilled 10% glycerol.

### Image acquisition and analysis

Growth conditions and staining procedures prior to microscopy are described in the figure legends. Prior to imaging, cells were immobilized on 2% agarose pads containing the appropriate growth medium, and covered with #1.5 coverslips [58]. Images were cropped and adjusted using FIJI software [59].

Microscopy: Images were obtained using a Nikon Ti inverted microscope that is fitted with a Nikon motorized stage with an OkoLab gas incubator with a slide insert attachment, an Andor Zyla 4.2 Plus sCMOS camera, Lumencore SpectraX LED Illumination, Plan Apo lambda 100x/1.45 Oil Ph3 DM objective lens, and Nikon Elements 4.30 acquisition software. Images in the green and red channels were taken using Chroma 49002 and 49008 filter cubes, respectively. The microscope was maintained at 30°C using a custom-made environmental control chamber.

Microfluidic time-lapsed experiment: Specific experimental setups are described in the figure legends. Cells were loaded into the CellASIC Onix B04 microfluidic plates (Millipore Sigma) that were attached to the microscope described above using using a multi-well insert. Each imaging chamber was flushed with the appropriate growth medium before loading the cells. During the course of the time-lapse, appropriate growth media were supplied to the cells using a constant flow rate of 60 psi. To avoid reduction of growth rate due to phototoxicity, we reduced the intensity of the excitation light using neutral density filters (at least ND8) in all our experiments.

Single-cell image analysis: Measurements of cell lengths and fluorescence signals at the single cell level were carried out using Oufti [60]. To identify cells with septal signals, the Oufti output was additionally analyzed using peakFinder, which is an add-on from Oufti, to identify the position of fluorescence peaks along the length of the cells. Custom-written Matlab code was used to further identify cells with a fluorescence peak that is located within 0.3–0.7 position within the normalized length of the cell. Plots of cummulative percentage of cells with a septal signal as a function of cell length were generated in MATLAB. The curves were fitted with the Hill coefficient using MATLAB as described in the figure legend.

Demograph generation: Using custom-written MATLAB code, cells were arranged from top to bottom according to their cell lengths. Additionally, in demographs of both the bait and prey, each cell was oriented such that the cell pole with the higher *bait* intensity was located on the right.

Correlation of bait and prey signal distribution between the cell poles: Only signals within 780 nm from the cell poles were used for the analysis. We first calculated the fractional difference in the bait signal between the left and right poles as designated in the demograph, using the equation $d_{bait}=\frac{B_{L}-B_{R}}{0.5\times (B_{L}+B_{R})}$, in which *B*_*L*_ and *B*_*R*_ represents the bait signals at the left and the right cell pole, respectively. The polar prey signals were analyzed similarly using the equation $d_{prey}=\frac{P_{L}-P_{R}}{0.5\times (P_{L}+P_{R})}$, in which *P*_*L*_ and *P*_*R*_ represents the bait signals at the left and the right cell pole, respectively. Finally, the correlation index, CI, was calculated using the equation $CI=\frac{d_{prey}}{d_{bait}}$ (*CI* = 1: complete positive correlation, *CI* = 0: no correlation and *CI* = -1: complete negative correlation).

### Fluorescent measurement of SteA or SteB protein levels

Overnight cultures of cells expressing mScar fusions to SteA, SteA variants, or SteB were diluted 1:1000 in fresh BHI and incubated shaking at 30°C until the optical density reached 0.5. An aliquot of cells (25 mL) was harvested by centrifugation and resuspended in 100 μL Lysis Buffer A (20 mM Tris 7.5, 10 mM EDTA, 1 mg/ml lysozyme, 1 mM PMSF, 10 *μ*g/ml DNAseI and 100 *μ*g/ml RNAseA). The cell suspensions were incubated at 37°C for 30 min before an equal volume of 2x SDS loading buffer was added. The samples were sonicated for 10 min to reduce the viscosity before they were resolved on a 4–20% Criterion TGX Precast Midi Protein Gel (Biorad). The gel was washed in H_2_O for 10 min before it was imaged with the Cy3 and Cy5 channels on a Typhoon FLA Scanner (GE Healthcare). The image was scaled and cropped using FIJI [59].

### Phylogenetic tree

For the phylogenetic tree showing the distribution of SteA, SteB and RecA homologs, the amino acid sequences of SteA (cgp_1603), SteB (cgp_1604) and RecA (cgp_2141) were used as a query in a BLASTp search against the NCBI “non redundant” (*nr*) database [61] with an *e*-value cutoff of 1e^-4^ for each protein. A list of all the taxa for which significant BLAST results were found was then sorted. We used a complex and diverse set of 1773 bacterial taxa called “Representative Genomes” that is available on NCBI (ftp://ftp.ncbi.nlm.nih.gov/blast/db/, Representative_Genomes.00.tar.gz). The phylogenetic tree was constructed using PhyloT (http://phylot.biobyte.de/) and BLASTp results were plotted against the tree. The tree was visualized and annotated using iToL (http://itol.embl.de/) [62].

### Genetic neighborhood analysis

A database of 1542 fully assembled representative bacterial genomes was created (https://www.ncbi.nlm.nih.gov/assembly). The same queries as for the phylogenetic tree were used in a tBLASTn against this database. The start and stop positions of the alignment were sorted for each genome and the distances between *steA* and *steB* or *steA* and *recA* were calculated for each organism.

## Supporting information

S1 Text Description of plasmids construction. (PDF)

S1 Table Summary of MB001 gene essentiality analysis. (XLSX)

S2 Table Comparison of essential genes in *Mycobacterium tuberculosis* to their homologs in *Corynebacterium glutamicum*. (XLSX)

S3 Table List of *ste* candidates. (XLSX)

S4 Table Strains used in this study. (PDF)

S5 Table Plasmids used in this study. (PDF)

S1 Fig Identification of essential genes in *Cglu* by Tn-seq. (**A**) Histogram showing the transposon insertion frequency (number of transposon insertion reads per gene base pair) for all genes in the MB001 genome. The overlaid black curve marks the Gaussian distribution fit. The blue dashed line (two standard deviations from the mean of the Gaussian fit) defines essential genes (red bars) from nonessential genes (grey bars). (**B**) Occurrence of essential genes in *Cglu* is constant across gene length, except for genes shorter than 100 bp. Gene essentially was characterized for every single gene using the criteria described in (**A**). All genes were binned at 100 bp intervals except for the last bin (which contains all genes larger than 2001 bp). Percentage of essential genes for each bin was plotted.
(TIF)

S2 Fig Comparison of the FtsW proteins from *E*. *coli*, *B*. *subtilis*, *Mtb* and *C*. *glutamicum* by multiple sequence alignment (MSA). MSA of the sequences was carried out using the T-coffee MSA server [63,64]. The output was then displayed using the BoxShade program. Sources of the FtsW protein sequences: *E*. *coli* (K-12), *B*. *subtilis* (168), *Mtb* (H37Rv) and *C*. *glutamicum* (MB001).
(TIF)

S3 Fig Screening for and validation of *ste* mutants. (**A**) Volcano plot showing the ratio of sequencing reads of each gene after growing the mutant library in growth medium supplemented with or without EMB compared to the p-value from Mann-Whitney *U*. Each filled circle in the plot represents a unique non-essential *Cglu* gene. Circles that fall in the area shaded yellow had at least 3-fold reduced sequencing reads in the presence of EMB and a p-val lower than 0.05 and were therefore categorized as *ste* genes. (**B**) Overnight cultures of MB001 (WT) and its indicated derivatives were normalized to an OD_600_ of 0.5, serially diluted, and spotted (5 μl) onto BHI agar medium with and without 1 μg/ml EMB as indicated. Plates were incubated for 24 hours at 30°C and photographed. Note that mutants forming aggregates in solution were vortexed for 3 seconds to resuspend the cells before OD_600_ measurements were taken for normalization.
(TIF)

S4 Fig Phylogenetic distribution of SteA and SteB proteins. (**A**) Shown is a phylogenetic tree depicting the occurrence of SteA (green), SteB (dark blue) and RecA (light blue) proteins as indicated by the colored regions at the outer edge of the tree. The tree was constructed in PhyLoT (http://phylot.biobyte.de) and visualized in iTOL [62] with a diversity set of 1773 strains. RecA occurrence serves as a control. Names of relevant bacterial orders or families are indicated in the tree. (**B**) *steA-steB* gene linkage. Histogram showing the genetic distance between 189 *steA* loci (green) and the nearest *steB* or *recA* locus (dark and light blue, respectively). If both genes are present, the distance is measured between the asterisks (from the middle of the *steA* gene to the middle of the other gene). When both genes are present, *steA* loci are commonly observed in an apparent operon with *steB*. Distances between *steA* and the nearest *recA* gene are shown in light blue as a negative control.
(TIF)

S5 Fig Correction of *ste* inactivation phenotype by ectopic gene expression. Spot dilutions of MB001 (WT) and the indicated derivatives: *ΔsteA* (HL2), *ΔsteB* (HL6) and *ΔsteA ΔsteB* (HL4). The control vector (pK-PIM) and constructs encoding *steA* (pHCL57), *steB* (pHCL59) and the *steAB* operon (pHCL58) under the P_*steA*_ promoter were integrated in the genome of the indicated strains. Overnight cultures of the indicated strains were normalized to OD_600_ of 0.5, serially diluted, and spotted (5 μl) onto BHI agar medium with and without 0.75 μg/ml EMB as indicated. Plates were incubated for 30 hours at 30°C and photographed.
(TIF)

S6 Fig RipA inactivation exacerbates the cell separation defect of *ΔripC* cells. Images of mutants lacking *ripA* (HL8) or *ripC* (HL7) or both (HL9). The mutant lacking both genes showed more severe cell separation phenotypes than mutants devoid of only one of those genes, confirming a previously published result [27]. Overnight cultures of the indicated strains were diluted 1:1000 and grown in BHI medium at 30°C. When OD_600_ of the cultures reached 0.2–0.3, cells were stained with FM 4–64 (1.5 μg/ml) for 5 min, spotted directly on an agarose pad and imaged by fluorescence microscopy.
(TIF)

S7 Fig Functional analysis of mScar-SteA and mScar-SteB. Histograms showing cell length distributions of MB001 (WT) and the indicated derivatives. Both mScar-SteA and mScar-SteB were produced from genome integrated plasmids under P_*steA*_ control in the *ΔsteA* mutant (HL2) and the *ΔsteB* mutant (HL6), respectively. Overnight cultures were diluted 1:1000 in BHI and grown at 30°C. When the OD_600_ reached 0.2–0.3, cells were diluted 10-fold and loaded into a CELLASIC ONIX microfluidic device for phase-contrast microscopy. (**A** & **B**) Cells were automatically detected from phase-contrast images using Oufti [60]. Cell lengths were calculated from cell outlines using MATLAB. (**C**) Phase-contrast images of the indicated strains from (**B**). Scale bars, 3 μm. (**D**) Overnight cultures of the indicated strains from (**B**) were normalized to an OD_600_ of 0.5, serially diluted, and spotted (5 μl) onto BHI agar medium with and without 1 μg/ml EMB as indicated. Plates were incubated for 24 hours at 30°C and photographed. Note that mutants with cell separation defects that form aggregates were vortexed for 3 seconds to resuspend cells before OD_600_ measurements were taken for normalization.
(TIF)

S8 Fig mScar-tagged full-length SteA and its variants are expressed at similar levels in the *ΔsteA* mutant. (**A**) SDS-page gel showing expression levels of the indicated mScar-tagged SteA variants in the *ΔsteA* mutant. Constructs encoding the indicated mScar-SteA variants under P_*steA*_ control were integrated in the genome of the *ΔsteA* mutant (HL2). Overnight cultures of the indicated strains were diluted to 1:1000 and grown in BHI medium at 30°C. Cells were harvested at OD_600_ ~ 0.5, resuspended in Buffer A and lysed by lysozyme treatment and sonication. Cell extracts were mixed with SDS loading buffer and resolved on a Pre-cast Criterion TGX gel (Biorad). mScar-fused SteA variants were detected using the Cy3 emission filter, by taking advantage of the intrinsic fluorescence of mScar. Bands in the ladder were revealed using a combination of Cy3 and Cy5 emission filters. Constructs encoding the indicated mScar-SteA variants are as follows: pHCL171: SteA; pHCL172: SteA_N_; pHCL173: SteA_N_-TM_2_; pHCL174: SteA_N_-TM_2_; pHCL175: mScar-TM_A_.
(TIF)

S9 Fig Subcellular localization of SteA and SteA variants. Representative images of the indicated strains from **S8 Fig**. Overnight cultures were diluted 1:1000 in BHI and grown at 30°C. When the OD_600_ reached 0.2–0.3, cells were diluted 10-fold and loaded on the CELLASIC ONIX microfluidic device for phase-contrast and fluorescence microscopy. Scale bar, 2 μm.
(TIF)

S10 Fig mScar-tagged full-length SteA is expressed at similar levels in the indicated deletion mutants. mScar-SteA was produced under P_*steA*_ control from pHCL171, which was integrated in the genome of the relevant mutants (HL2: *ΔsteA*, HL4: *ΔsteA ΔsteB*, HL10: *ΔsteA ΔripC* and HL16: *ΔsteA ΔftsEX*). Overnight cultures of the indicated strains were diluted to 1:1000 and grown in BHI medium at 30°C. Cells were harvested at OD_600_ ~ 0.5, resuspended in Buffer A and lysed by lysozyme treatment and sonication. Cell extracts were mixed with SDS loading buffer and resolved on a Pre-cast Criterion TGX gel (Biorad). mScar-fused SteA variants were detected using the Cy3 emission filter, by taking advantage of the intrinsic fluorescence of mScar. Bands in the ladder were revealed using a combination of Cy3 and Cy5 emission filters.
(TIF)

S11 Fig Examination of protein-protein interactions using the POLAR two-hybrid assay. (**A, C** & **D**) *Top row*: representative fluorescence images of TB28 *E*. *coli* cells expressing the indicated the bait and prey proteins. Cells were grown for imaging as in **Fig 9B**. The indicated baits were expressed using the following plasmids: pHCL149 (TM control), pHCL202 (SteA), pHCL204 (SteAB) and pHCL205 (FtsEX). The preys were expressed from genome integrated plasmids (pHCL194: SteB, pHCL196: SteB_N_, pHCL214: SteA and pHCL225: RipC_N_). *Bottom rows*: demographs showing fluorescence distribution of the corresponding preys and baits at the population level. Single-cell fluorescence quantification was performed using Oufti [60]. Cells were oriented using a custom-written MATLAB script such that the cell pole with the higher bait fluorescence was located on the right of the demograph. At least 250 cells were used to generate each demograph. (**B**) Quantification of fluorescence correlation between the bait-prey pairs at the cell pole was performed as described in **Fig 10A**.
(TIF)

S12 Fig *Cglu* RipC has an extended N-terminal domain compared to two other FtsX-interacting proteins, PcsB and EnvC. Scaled domain representations of PcsB (spd_2043), EnvC (b3613) and RipC (cgp_1735). The coiled coil region of PcsB was identified from a solved X-ray crystal structure of the protein (PDB ID: 4CGK) [65]. Coiled coil regions RipC and EnvC were predicted using the algorithm of Lupas *et al*. [66]. The corresponding PG hydrolase domain of each protein was identified using Pfam [67]. The signal sequence of each protein was identified using the Phobius algorithm [68] and excluded from the cartoon representation.
(TIFF)
