# Supplementary material for: Identification of new components of the RipC-FtsEX cell separation pathway of Corynebacterineae
Source: PLoS Genet. 2019 Aug 22;15(8):e1008284. doi: 10.1371/journal.pgen.1008284 (PMC6705760; doi:10.1371/journal.pgen.1008284)
Supplement: S1 Text — (PDF) [file pgen.1008284.s001.pdf]

S1 Text. Plasmid construction methods

pHCL41: Two DNA fragments corresponding to sequences downstream and upstream of cgp_1603 (*steA*) were amplified using the following sets of primers: 1) AAAAGGATATCAATACGTCCTGCAACCACCAC & ACCGCAAAGCCGACAAAtagGAAGGCAACATGGCTAAAC and 2) TAATCTCTAGACAGCTCGCAGCAAATGCCTTTG & ATGTTGCCTTCctaTTTGTCGGCTTTGCGGTTGAACAG, using MB001 gDNA as the template. These fragments were spliced together by overlapping extension PCR. The resulting fragment was digested with XbaI and EcoRV and subsequently ligated with similarly digested pCRD206.

pHCL42: Two DNA fragments corresponding to sequences upstream of of *steA* and downstream of *steB* (cgp_1604) were amplified using the following sets of primers: 1) TAATCTCTAGACAGCTCGCAGCAAATGCCTTTG & GCTTGGAAAACctaCTGGTCGGCTTTGCGGTTGAAC and 2) AATTGGATATCCAGGTACTCTCCCCTGCGCTC & ACCGCAAAGCCGACCAGtagGTTTTCCAAGCCTTTAAAACG, and MB001 gDNA as the template. These fragments were spliced together by overlapping extension PCR. The resulting fragment was digested with XbaI and EcoRV and subsequently ligated with similarly digested pCRD206.

pHCL46: Two DNA fragments corresponding to sequences upstream and downstream of *steB* were amplified using the following sets of primers: 1) AAAACTCTAGAGGCAACACCATTCAGTTCATTCAC & AAAACCTACTGCGCTCTTCCACGTCGTTTAGCCATG and 2) TAATGGATATCCCAGGTACTCTCCCCTGCGCTC & AACGACGTGGAAGAGCGCAGTAGGTTTTCCAAGC, and MB001 gDNA as the template. These fragments were spliced together by overlapping extension PCR. The resulting fragment was digested with XbaI and EcoRV and subsequently ligated with similarly digested pCRD206.

pHCL54: Two DNA fragments corresponding to sequences upstream of *ftsE* (cgp_0914) and downstream of *ftsX* (cgp_0915) were amplified using two pairs of primers: 1) ATAAATCCTGGTGTCCCTGTTGGATACGGCGACTACATGTACGG & CACGTAGAAGCGCAGGGTGATcacGGGTCACACTTTAG and 2) GACCCgtgATCACCCTGCGCTTCTACGTGAGGAAAtaaG & CAAGCTTGCATGCCTGCAGGTCGACTGCCAGAGGTCTGGCTCAAGG, and MB001 gDNA as the template. These fragments were subsequently ligated with the vector which was amplified using primers (AGTCGACCTGCAGGCATGCAAGCTTGGCAC & ATCCAACAGGGACACCAGGATTTATTTATTC) and pCRD206 as the template, by Gibson assembly.

pHCL57: The insert encoding P_{steA1}-SteA was amplified using MB001 gDNA as the template and the following primers: AGCTCGAGCTCTTAATTAACGTTAAGGAGCTGCTCGAGCGTGC & TGCGGCCGCCGCGGGGCCCAGATCTActaTTTGAACCAACCCTGGACTG, and ligated with SpeI-digested pK-PIM by Gibson assembly.

pHCL58: The insert encoding P_{steA1}-SteAB was amplified using MB001 gDNA as the template and the following primers: AGCTCGAGCTCTTAATTAACGTTAAGGAGCTGCTCGAGCGTGC &

TGCGGCCGCCGCGGGGCCCAGATCTActaCTGCGCTGGCGCTGC, and ligated with SpeI-digested pK-PIM by Gibson assembly.

pHCL59: DNA sequence corresponding to *steA* was deleted from pHCL58 by inverse PCR using **pHCL58** as the template and catGCAGCCCATTGTGGAAC & TGGAACAGCTTCGCGCTGAC that have been phosphorylated at their ends as the primers. The resulting DNA fragment was the circularized by end ligation.

pHCL66: Two DNA fragments corresponding to sequences upstream and downstream of *ripA* (cgp_2402) were amplified using two primer pairs: 1) ATAAATCCTGGTGTCCCTGTTGGAGGAGTGGCGTAGAGCCTGTAG & AGTGGAATGGCATcacTGATGAAACTCCAAATTCTTCCTG and 2) GAGTTTCATCAgtgATGCCATTCCACTCTGCAGTTC & CAAGCTTGCATGCCTGCAGGTCGACTTGTGGCGTCAGGGCATCC, and MB001 gDNA as the template. The vector was amplified using AGTCGACCTGCAGGCATGCAAGCTTGGCAC & ATCCAACAGGGACACCAGGATTTATTTATTC as the primers and pCRD206 as the template. The fragments were ligated with the vector by Gibson assembly.

pHCL67: Two DNA fragments corresponding to sequences upstream and downstream of *ripC* (cgp_1735) were amplified using two sets of primers: 1) ATAAATCCTGGTGTCCCTGTTGGACTCCAATGTCGGCTGCGGTG & CTCTCGGTCATTCCcacATGTTCTCCTGTTCAATCCG and 2) CACAGGAGAACATgtgGGAATGACCGAGAGCGTGGTAC & CAAGCTTGCATGCCTGCAGGTCGACTGACACACCAACAGCCTCAGC, and MB001 gDNA as the template. The vector was amplified using AGTCGACCTGCAGGCATGCAAGCTTGGCAC & ATCCAACAGGGACACCAGGATTTATTTATTC as the primers and pCRD206 as the template. The fragments were ligated with the vector by Gibson assembly.

pHCL84: Two DNA fragments corresponding to sequences the upstream and downstream of cgp_2888 were amplified using two sets of primers: 1) ATAAATCCTGGTGTCCCTGTTGGATCACAGAAGACGATGTCGAGC & TttaGCTACGTGGGGTGCGCAGAACGATTTGTCCGTCAGACTGGTTG and 2) gGACAACCAGTCTGACGGACAAATCGTTCTGCGCACCCCACGTAG & AAGCTTGCATGCCTGCAGGTCGACTATTGAGGGCATTCGACAGCTG, and MB001 gDNA as the template. The vector was amplified using AGTCGACCTGCAGGCATGCAAGCTTGGCAC & ATCCAACAGGGACACCAGGATTTATTTATTC as the primers and pCRD206 as the template. The fragments were ligated with the vector by Gibson assembly.

pHCL85: Two DNA fragments corresponding to sequences upstream and downstream of cgp_2887 were amplified using two sets of primers: 1) ATAAATCCTGGTGTCCCTGTTGGATCTTATTGAAGCATTGGCGCAG & GCCCTTGATGCCTttaAGAAACCGCCGCAGCAACATAAGGATTTTCc and 2) TatgGAAAATCCTTATGTTGCTGCGGCGGTTTCTtaaAGGCATCAAG & AAGCTTGCATGCCTGCAGGTCGACTACGCTTGCTCTATGTCATGC, and MB001 gDNA as the template. The vector was amplified using AGTCGACCTGCAGGCATGCAAGCTTGGCAC & ATCCAACAGGGACACCAGGATTTATTTATTC as the primers and pCRD206 as the template. The fragments were ligated with the vector by Gibson assembly.

pHCL88: Two DNA fragments corresponding to sequences upstream and downstream of cgp_1836 were amplified using two primer sets: 1) ATAAATCCTGGTGTCCCTGTTGGATCGAGGCACTCTAAGCCTTCAG & aTCGGTTGCTGTCTAGAACGCCACTGCGGTTTCGGATTTGGTTcatC and 2) CAGGatgAACCAAATCCGAAACCGCAGTGGCGTTCTAGACAGCAAC & AAGCTTGCATGCCTGCAGGTCGACTGTCCGTAGGACTGGTGTGCCAG, using MB001 gDNA as the template. The vector was amplified using AGTCGACCTGCAGGCATGCAAGCTTGGCAC & ATCCAACAGGGACACCAGGATTTATTTATTC as the primers and pCRD206 as the template. The fragments were ligated with the vector by Gibson assembly.

pHCL106: SpeI- and NotI-digested pK-PIM was ligated with three DNA fragments: 1) SpeI- and BglII- digested $P_{ftsZ123}$ (amplified using MB001 gDNA as the template and TTAGACTAGTGTCTCCGGGATTGTTGAGGG & TATAGAGATCTcatTGTCGATGTCTCGCCTTTCG as the primers), 2) BglII- and EcoRI-digested gBlock encoding *msfGFP* and 3) EcoRI- and NotI-digested *ftsZ* (amplified using MB001 gDNA as the template and TTTTGGCGGCCGCttaCTGGAGGAAGCTGGGTAC & AAAGGAATTCACCTCACCGAACAACTACCTC as the primers).

pHCL131: SpeI- and NheI-digested pK-PIM was ligated with two DNA fragments: 1) SpeI- and BglII-digested P_{steA} (amplified using MB001 gDNA as the template and AAAGACTAGTGCACGTTGCCAAGAATGTAGG & AAAGAGATCTCATGCAGCCCATTGTGGAAC as the primers) and 2) BglII- and NheI-digested gBlock encoding *mScarlet-I* (*mScar*).

pHCL147: BamHI- and HindIII-digested pMT178 was ligated with the similarly digested gBlock insert encoding for ^{ss}*dsbA*-*mScarC*.

pHCL149: XbaI- and HindIII-digested pTB285 was ligated with the NheI- and HindIII-digested gBlock insert encoding for *popZ-rbs-H3H4-msfGFP-*tm_{ponB} to generate pHCL148. pHCL148 was digested with NotI and HindIII to remove the R6K *ori* and ligated with the similarly digested fragment harboring the ColE1 *ori* from pHC897.

pHCL170: NotI-digested pHCL131 was ligated with the insert, which was amplified from MB001 gDNA using CGTGGTTCGGAAGCTATTACCGCCGCTgctaaacgacgtggaagaggc & GTAAAACGACGGCCAGTGAATTCACGTGCtactgcgctggcgctgctg as the primers, by Gibson assembly.

pHCL171: NotI-digested pHCL131 was ligated with the insert, which was amplified from MB001 gDNA using CGTGGTTCGGAAGCTATTACCGCCGCTctgttcaaccgcaaagccgac & GTAAAACGACGGCCAGTGAATTCACGTGCtagaaccaaccctggactgtc as the primers, by Gibson assembly.

pHCL172: NotI-digested pHCL131 was ligated with the insert, which was amplified from MB001 gDNA using CGTGGTTCGGAAGCTATTACCGCCGCTctgttcaaccgcaaagccgac & GTAAAACGACGGCCAGTGAATTCACGTGCtagaaccaaccctggactgtc as the primers, by Gibson assembly.

pHCL173: NotI-digested pHCL131 was ligated with two DNA fragments: 1) *steA*$_N$, which was amplified from MB001 gDNA using GTGGTTCGGAAGCTATTACCGCCGCTTCCGTCATCGCAAGTCTTTACAC & tccaaggttgctggagctgc as the primers and 2) a gBlock encoding the C-terminal transmembrane peptide of *Streptomycetes coelicolor* SecE (SRNQLTTYTTVVIIFVVIMIGLVTLIDYGFSHAAKYVFG), by Gibson assembly.

pHCL174: NotI-digested pHCL131 was ligated with two DNA fragments: 1) *steA*$_N$, which was amplified from MB001 gDNA using GTGGTTCGGAAGCTATTACCGCCGCTTCCGTCATCGCAAGTCTTTACAC & tccaaggttgctggagctgc as the primers and 2) a gBlock encoding the C-terminal transmembrane peptide of *Streptomycetes coelicolor* PkaB (RRRRIAVGAGAVALVAAIGVGTWLATGGDEDGGGPQDTRNSAPAAP), by Gibson assembly.

pHCL175: NotI-digested pHCL131 was ligated with the insert, which was amplified from MB001 gDNA using GTGGTTCGGAAGCTATTACCGCCGCTTCCGTCATCGCAAGTCTTTACAC & GTAAAACGACGGCCAGTGAATTCACGTGCTAGAACCAACCCTGGACTGTC as the primers, by Gibson assembly.

pHCL194: XbaI- and BamHI-digested pHCL147 was ligated with the XbaI- and BglII-digested insert encoding *steB*, which amplified using MB001 gDNA as the template and GCTATCTAGATTTAAGAAGGAGATATACATatggctaaacgacgtggaagaggc & cgttagagatctctgcgctggcgctgctgcag as the primers.

pHCL195: The *ripC* gene was amplified from MB001 gDNA in two fragments to create the C543S mutation. Two primer sets: 1) AAACAGAGATCTCAGCCACAGAATCCGGATGAC & CAGTCCAGAGCTGTCGAAGCCAACCTTGTTGTAATCG and 2) CAACAAGGTTGGCTTCGACAGCTCTGGACTGACCTTGTATGC & AAAATCCTCGAGAATGAGGCGTACCACGCTCTC were used to generate two fragments that shared one homologous end harboring the desired mutation. The fragments were spliced together by overlapping extension PCR, digested with BglII and XhoI before ligating with BamHI- and XhoI-digested pHCL147.

pHCL196: Same as described for **pHCL194**, except that the insert was digested with XbaI and BamHI.

pHCL202: BamHI- and HindIII-digested pHCL149 was ligated with the *steA* insert, which was amplified using MB001 gDNA as the template and gaattgtataaaggttctggcggtggaagtctgttcaaccgcaaagcc & ttcgctcgcgtatcggtgatAAGCTTatttgaaccaaccctggactgtc as the primers, by Gibson assembly.

pHCL204: BamHI- and HindIII-digested pHCL149 was ligated with the *steAB* insert, which was amplified using MB001 gDNA as the template and gaattgtataaaggttctggcggtggaagtctgttcaaccgcaaagcc & gttcgctcgcgtatcggtgataagcttggaaaacctactgcgctggc as the primers, by Gibson assembly.

pHCL205: XhoI-digested pHCL150 (P$_{ara}$-*popZ-rbs-msfGFP-H3H4*) was ligated with the *ftsEX* insert, which was amplified using MB001 gDNA as the template and atttaagaaggagatatacatatgcTCATCACCTTCGAGAACGTCACC &

agaaccgccaccagagcctccacccgaTTTCCTCACGTAGAAGCGCAG as the primers, by Gibson assembly.

pHCL213: XhoI- and HindIII-digested pHCL149 was ligated with the similarly digested insert, which was amplified using MB001 gDNA as the template and as gcctcgagGGATCTCAGCCACAGAATCCGGATG and AAAGGAAGCTTAACGATCGCCGGAAATAACCTC the primers.

pHCL214: BamHI- and HindIII-digested pHCL152 (lacI-P_{lac}-*mscarN*) was ligated with the similarly digested gBlock encoding *steA* that has been codon-optimized for expression in *E. coli*.

pHCL225: BamHI- and HindIII-digested pHCL151 (lacI-P_{lac}-^{ss}*dsbA*-*mscarN*) was ligated with the BglII- and HindIII-digested insert, which was amplified using AAACAGAGATCTCAGCCACAGAATCCGGATGAC & AAAAAGaagcttaCTGAGCGGAACGATCGCCG as the primers and MB001 gDNA as the template.

pHCL269: The vector, which was amplified using AGTCGACCTGCAGGCATGCAAGCTTGGCAC & ATCCAACAGGGACACCAGGATTTATTTATTC as the primers and pCRD206 as the template, was ligated by Gibson assembly with a gBlock insert encoding DNA regions upstream and downstream of cgp_0575.
