# Supplementary material for: Identification of new components of the RipC-FtsEX cell separation pathway of Corynebacterineae
Source: PLoS Genet. 2019 Aug 22;15(8):e1008284. doi: 10.1371/journal.pgen.1008284 (PMC6705760; doi:10.1371/journal.pgen.1008284)
Supplement: S4 Table — (PDF) [file pgen.1008284.s005.pdf]

S4 Table. Strains used in this study

Strain	Genotype	Source/Reference[a]
Escherichia coli strains		
DH5α(λ*pir*)	*F– hsdR17 deoR recA1 endA1 phoA supE44 thi-1 gyrA96 relA1 Δ(lacZYA-argF)U169 ϕ80dlacZΔM15* (λpir)	Gibco BRL
S17-1(λ*pir*)	*recA thi pro hsdR- M+ RP4::2-Tc::Mu::Km Tn7 Tp*^{r} *Sm*^{r} (λpir)	de Lorenzo et al. 1993
TB28	MG1655 *ΔlacIZYA::frt*	Bernhardt and de Boer 2004
Corynebacterium glutamicum strains		
MB001	ATCC 13032 ΔCGP1 (cg1507-cg1524) ΔCGP2 (cg1746-cg1752) ΔCGP3 (cg1890-cg2071)	Baumgart et al. 2013
HL2	MB001 *ΔsteA*	MB001/pHCL41, 2XO, This work
HL4	MB001 *ΔsteA ΔsteB*	MB001/pHCL42, 2XO, This work
HL6	MB001 *ΔsteB*	MB001/pHCL46, 2XO, This work
HL7	MB001 *ΔripC*	MB001/pHCL67, 2XO, This work
HL8	MB001 *ΔripA*	MB001/pHCL66, 2XO, This work
HL9	MB001 *ΔripA ΔripC*	HL8/pHCL67, 2XO, This work
HL10	MB001 *ΔsteA ΔripC*	HL2/pHCL67, 2XO, This work
HL11	MB001 *ΔsteB ΔripC*	HL6/pHCL67, 2XO, This work
HL12	MB001 *ΔsteA ΔripA*	HL8/pHCL41, 2XO, This work
HL13	MB001 *ΔsteB ΔripA*	HL8/pHCL46, 2XO, This work
HL14	MB001 *ΔftsEX*	MB001/pHCL54, 2XO, This work
HL16	MB001 *ΔftsEX ΔsteA*	HL14/pHCL41, 2XO, This work
HL19	MB001 *Δcgp_1836*	MB001/pHCL88, 2XO, This work
HL20	MB001 *Δcgp_2287*	MB001/pHCL85, 2XO, This work
HL21	MB001 *Δcgp_2288*	MB001/pHCL84, 2XO, This work
HL36	MB001 *Δcgp_0575*	MB001/pHCL269 2XO, This work

[a]Marker-less in-frame deletions by double homologous cross-over (2XO) of a temperature-sensitive integrative plasmid are described using the shorthand: Parental strain/plasmid, 2XO. In all cases, plasmid loss was confirmed by selecting for Kan^S- and $sucrose^R$- clones. Deletion was confirmed by diagnostic PCR.

References:

1. de Lorenzo V, Cases I, Herrero M, Timmis KN. Early and late responses of TOL promoters to pathway inducers: identification of postexponential promoters in Pseudomonas putida with lacZ-tet bicistronic reporters. J Bacteriol. 1993 Nov;175(21):6902–7.

2. Bernhardt TG, de Boer PAJ. The Escherichia coli amidase AmiC is a periplasmic septal ring component exported via the twin-arginine transport pathway. Mol Microbiol. NIH Public Access; 2003 Jun;48(5):1171–82.

3. Baumgart M, Unthan S, Rückert C, Sivalingam J, Grünberger A, Kalinowski J, et al. Construction of a prophage-free variant of Corynebacterium glutamicum ATCC 13032 for use as a platform strain for basic research and industrial biotechnology. Appl Environ Microbiol. American Society for Microbiology; 2013 Oct;79(19):6006–15.
