# Supplementary material for: Identification of new components of the RipC-FtsEX cell separation pathway of Corynebacterineae
Source: PLoS Genet. 2019 Aug 22;15(8):e1008284. doi: 10.1371/journal.pgen.1008284 (PMC6705760; doi:10.1371/journal.pgen.1008284)
Supplement: S5 Table — (PDF) [file pgen.1008284.s006.pdf]

S5 Table. Plasmids used in this study

Plasmid	Description	Source or Reference
Corynebacterium glutamicum (*Cglu*) plasmids		
pHCL41	Kan^R, pCRD206 derivative containing an insert covering upstream and downstream of *steA* (cgp_1603).	This study
pHCL42	Kan^R, pCRD206 derivative containing an insert covering upstream and downstream of *steAB* (cgp_1603 & cgp_1604).	This study
pHCL46	Kan^R, pCRD206 derivative containing an insert covering upstream and downstream of *steB* (cgp_1604).	This study
pHCL54	Kan^R, pCRD206 derivative containing an insert covering upstream and downstream of *ftsEX* (cgp_0914 & cgp_0915).	This study
pHCL57	Kan^R, pK-PIM derivative containing a P_{steA1}-steA.	This study [a]
pHCL58	Kan^R, pK-PIM derivative containing a P_{steA1}-steAB.	This study [a]
pHCL59	Kan^R, pK-PIM derivative containing a P_{steA2}-steB.	This study [b]
pHCL66	Kan^R, pCRD206 derivative containing an insert covering upstream and downstream of *ripA* (cgp_2402).	This study
pHCL67	Kan^R, pCRD206 derivative containing an insert covering upstream and downstream of *ripC* (cgp_1735).	This study
pHCL84	Kan^R, pCRD206 derivative containing an insert covering upstream and downstream of cgp_2888.	This study
pHCL85	Kan^R, pCRD206 derivative containing an insert covering upstream and downstream of cgp_2887.	This study
pHCL88	Kan^R, pCRD206 derivative containing an insert covering upstream and downstream of cgp_1836.	This study
pHCL106	Kan^R, pK-PIM derivative containing the $P_{ftsZ123}$-*msfGFP-ftsZ* insert.	This study [c]
pHCL131	Kan^R, pK-PIM derivative containing the P_{steA}-*mScarN* insert.	This study [d]
pHCL170	Kan^R, pK-PIM derivative containing the P_{steA}-*mScar-steB* insert.	This study [d]
pHCL171	Kan^R, pK-PIM derivative containing the P_{steA}-*mScar-steA* insert.	This study [d]
pHCL172	Kan^R, pK-PIM derivative containing the P_{steA}-*mScar-*$steA_N$ insert.	This study [d]
pHCL173	Kan^R, pK-PIM derivative encoding P_{steA}-*mScar-*$steA_N$-TM_1.	This study [d]
pHCL174	Kan^R, pK-PIM derivative encoding P_{steA}-*mScar-*$steA_N$-TM_2.	This study [d]

Plasmid	Description	Source or Reference
pHCL175	Kan^R, pK-PIM derivative encoding P_{steA}-*mScar-TM*$_A$.	This study [d]
pHCL269	Kan^R, pCRD206 derivative containing an insert covering upstream and downstream of cgp_0575.	This study
Escherichia coli plasmids		
pAH69	Kan^R, temperature-sensitive replicative plasmid encoding PR::int(HK022).	Haldiman and Wanner 2001
pHCL147	Tet^R, lacI-P_{lac}-^{ss}*dsbA-mscarC*.	Lim, HC [e, f]
pHCL149	Cm^R, P_{ara}-*popZ-rbs-H3H4-msfGFP-tm*$_{ponB}$.	Lim, HC
pHCL194	Tet^R, lacI-P_{lac}-*steB-mscar*.	This study
pHCL195	Tet^R, lacI-P_{lac}-*ripC(C543S)-mscar*.	This study
pHCL196	Tet^R, lacI-P_{lac}-*steB*$_N$-*mscar*.	This study
pHCL202	P_{ara}-*popZ-rbs-H3H4-msfGFP-steA*.	This study
pHCL204	P_{ara}-*popZ-rbs-H3H4-msfGFP-steAB*.	This study
pHCL205	P_{ara}-*popZ-rbs-ftsEX-msfGFP-H3H4*.	This study
pHCL213	P_{ara}-*popZ-rbs-H3H4-msfGFP-ripC*$_{50-491}$.	This study
pHCL214	Tet^R, lacI-P_{lac}-*mscar-steA*.	This study
pHCL225	Tet^R, lacI-P_{lac}-*mscar-ripC*$_{50-494}$.	This study

[a] P_{steA1} includes the DNA sequence 150 bp upstream of the *steA* gene. [b] P_{steA2} additionally includes 51 nt upstream of *steB* to include the native ribosomal binding site of *steB*, which is located at end of *steA*. As a result, this construct also produces a 14 amino acid peptide resembling the end of SteA. [c] $P_{ftsZ123}$ is the optimal promoter of the *ftsZ* gene as defined by Letek M. *et al.* 2007. [d] P_{steA} includes the DNA sequence 285 bp upstream of the *steA* gene. [e] ^{ss}*dsbA* corresponds to the first 24 codons of *E. coli dsbA* encoding its export signal to the periplasm. [f] *mscarC* indicates that the position of the linker and cloning sites have been optimized to facilitate the cloning of C-terminal *mscar* fusions. Numbers in subscript indicate the amino acids included in the relevant clones.

Unless indicated, the ribosome binding site (RBS) used for all *E. coli* constructs is the strong RBS of phage T7 *Φ*10 gene.

References:

1. Haldimann A, Wanner BL. Conditional-Replication, Integration, Excision, and Retrieval Plasmid-Host Systems for Gene Structure-Function Studies of Bacteria. J Bacteriol. 2001 Nov 1;183(21):6384–93.

2. Letek M, Ordóñez E, Fiuza M, Honrubia-Marcos P, Vaquera J, Gil JA, et al. Characterization of the promoter region of ftsZ from Corynebacterium glutamicum and controlled overexpression of FtsZ. Int Microbiol. 2007 Dec;10(4): 271–82.
