# Supplementary figures and images for: Identification of new components of the RipC-FtsEX cell separation pathway of Corynebacterineae
Source: PLoS Genet. 2019 Aug 22;15(8):e1008284. doi: 10.1371/journal.pgen.1008284 (PMC6705760; doi:10.1371/journal.pgen.1008284)

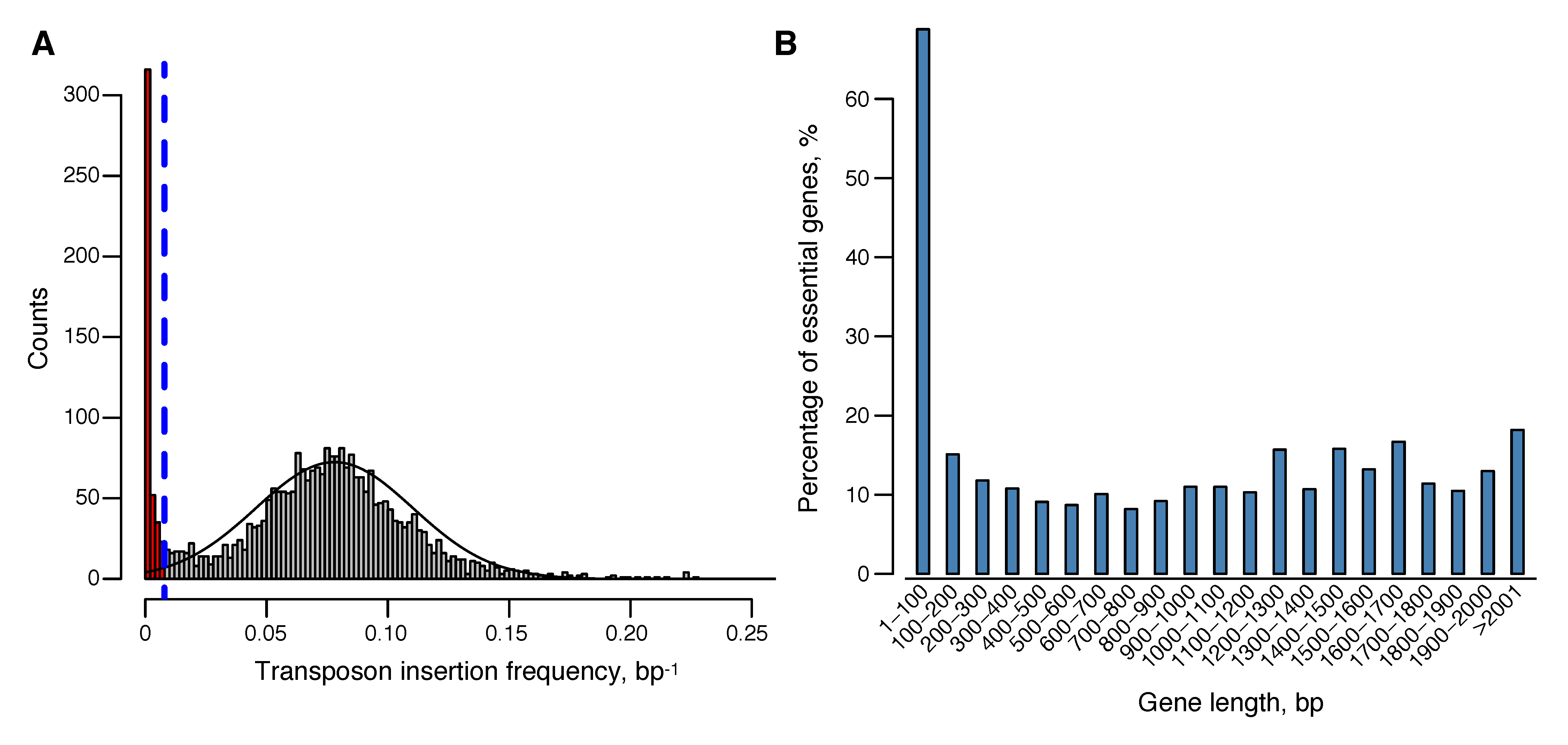

Supplement: S1 Fig — (A) Histogram showing the transposon insertion frequency (number of transposon insertion reads per gene base pair) for all genes in the MB001 genome. The overlaid black curve marks the Gaussian distribution fit. The blue dashed line (two standard deviations from the mean of the Gaussian fit) defines essential genes (red bars) from nonessential genes (grey bars). (B) Occurrence of essential genes in Cglu is constant across gene length, except for genes shorter than 100 bp. Gene essentially was characterized for every single gene using the criteria described in (A). All genes were binned at 100 bp intervals except for the last bin (which contains all genes larger than 2001 bp). Percentage of essential genes for each bin was plotted. (TIF) [file pgen.1008284.s007.tif]

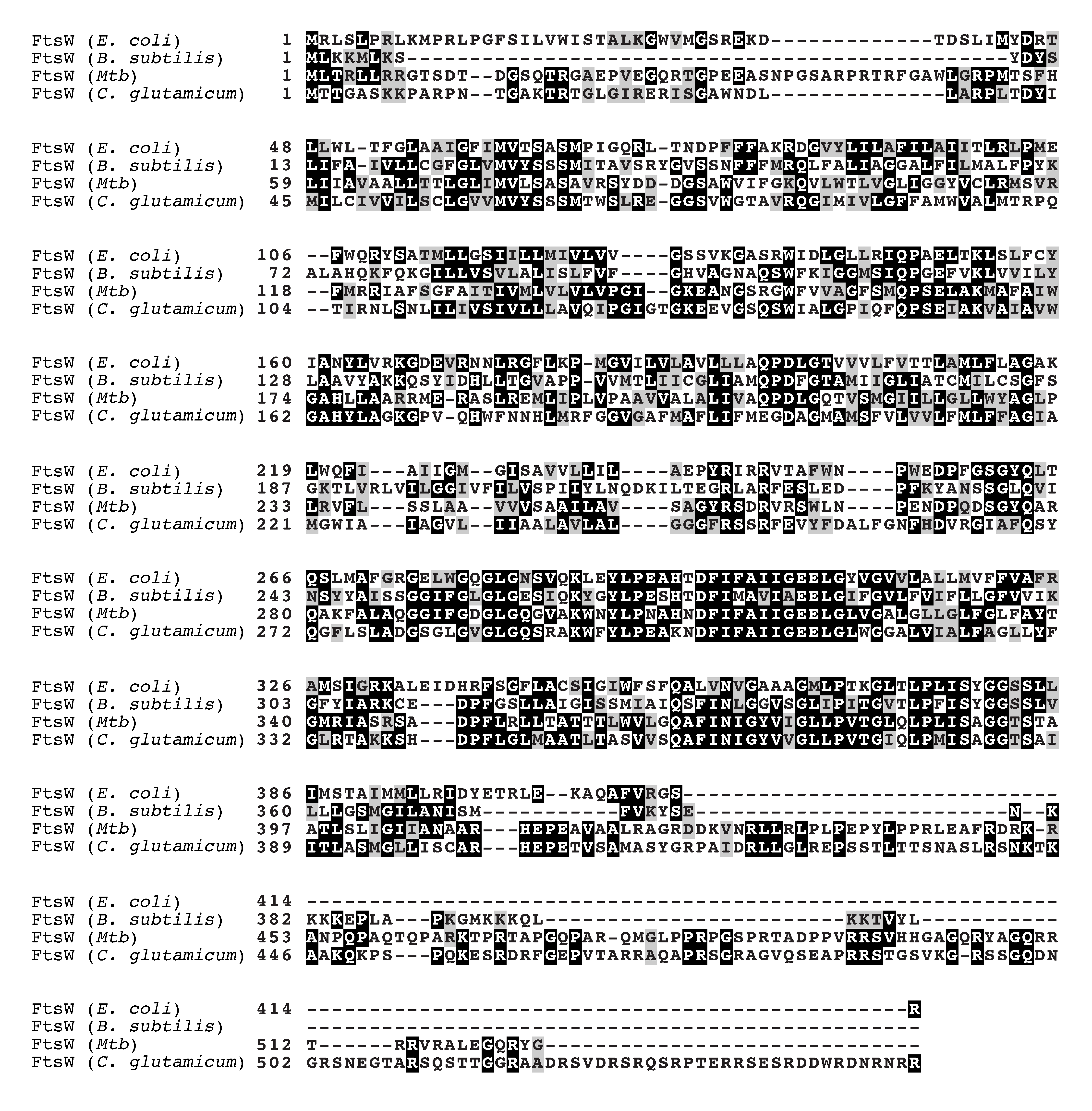

Supplement: S2 Fig — MSA of the sequences was carried out using the T-coffee MSA server [63,64]. The output was then displayed using the BoxShade program. Sources of the FtsW protein sequences: E. coli (K-12), B. subtilis (168), Mtb (H37Rv) and C. glutamicum (MB001). (TIF) [file pgen.1008284.s008.tif]

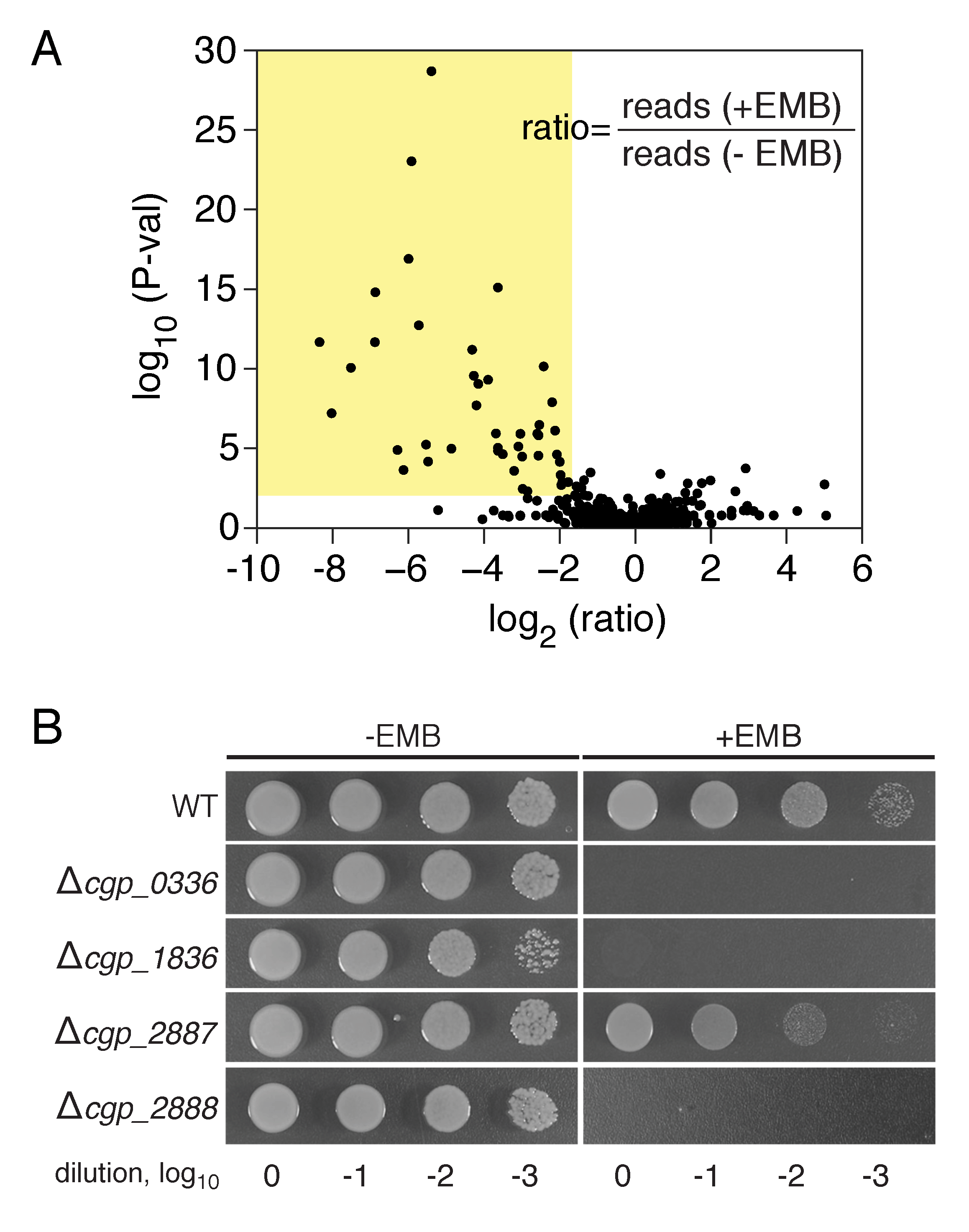

Supplement: S3 Fig — (A) Volcano plot showing the ratio of sequencing reads of each gene after growing the mutant library in growth medium supplemented with or without EMB compared to the p-value from Mann-Whitney U. Each filled circle in the plot represents a unique non-essential Cglu gene. Circles that fall in the area shaded yellow had at least 3-fold reduced sequencing reads in the presence of EMB and a p-val lower than 0.05 and were therefore categorized as ste genes. (B) Overnight cultures of MB001 (WT) and its indicated derivatives were normalized to an OD600 of 0.5, serially diluted, and spotted (5 μl) onto BHI agar medium with and without 1 μg/ml EMB as indicated. Plates were incubated for 24 hours at 30°C and photographed. Note that mutants forming aggregates in solution were vortexed for 3 seconds to resuspend the cells before OD600 measurements were taken for normalization. (TIF) [file pgen.1008284.s009.tif]

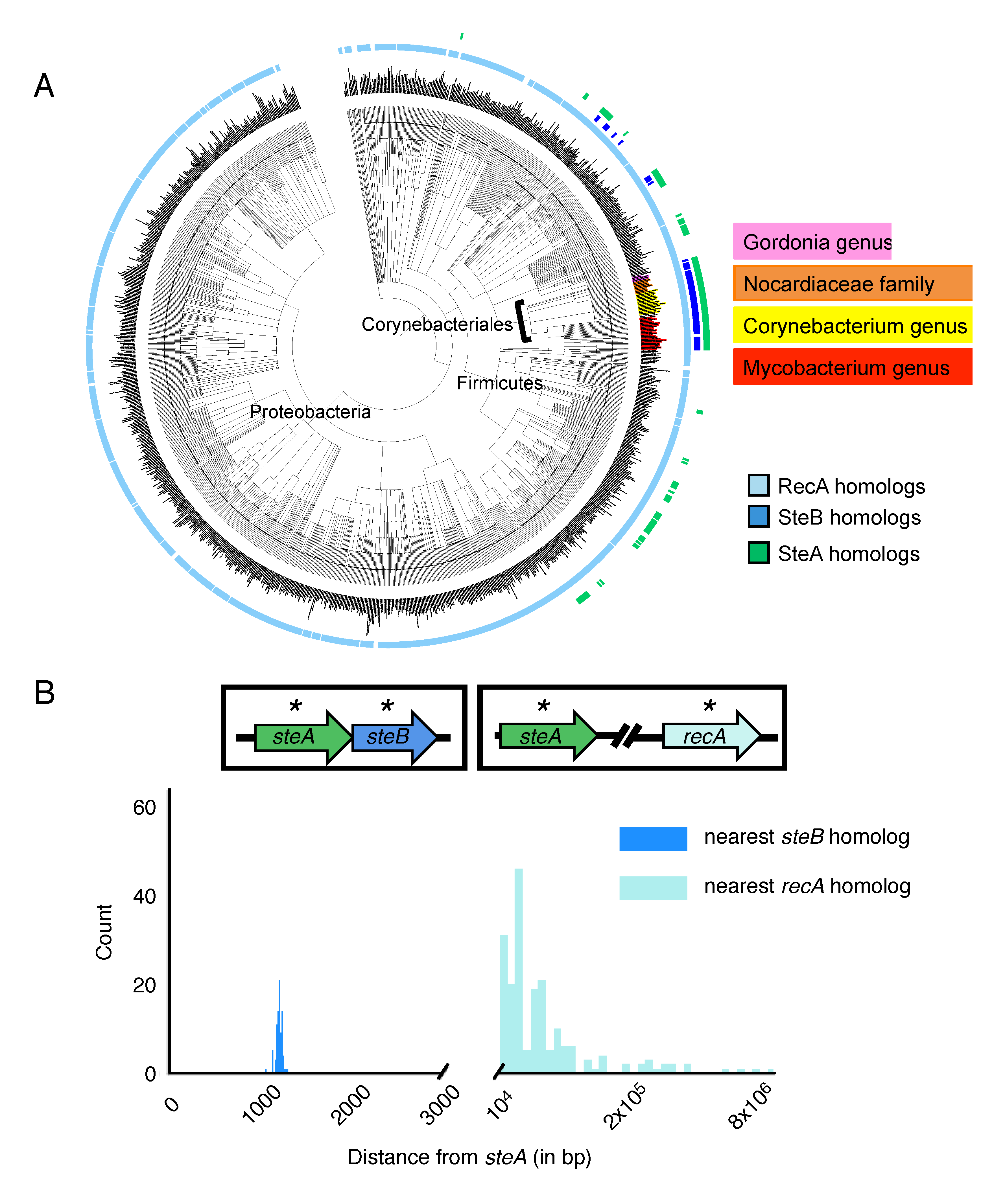

Supplement: S4 Fig — (A) Shown is a phylogenetic tree depicting the occurrence of SteA (green), SteB (dark blue) and RecA (light blue) proteins as indicated by the colored regions at the outer edge of the tree. The tree was constructed in PhyLoT (http://phylot.biobyte.de) and visualized in iTOL [62] with a diversity set of 1773 strains. RecA occurrence serves as a control. Names of relevant bacterial orders or families are indicated in the tree. (B) steA-steB gene linkage. Histogram showing the genetic distance between 189 steA loci (green) and the nearest steB or recA locus (dark and light blue, respectively). If both genes are present, the distance is measured between the asterisks (from the middle of the steA gene to the middle of the other gene). When both genes are present, steA loci are commonly observed in an apparent operon with steB. Distances between steA and the nearest recA gene are shown in light blue as a negative control. (TIF) [file pgen.1008284.s010.tif]

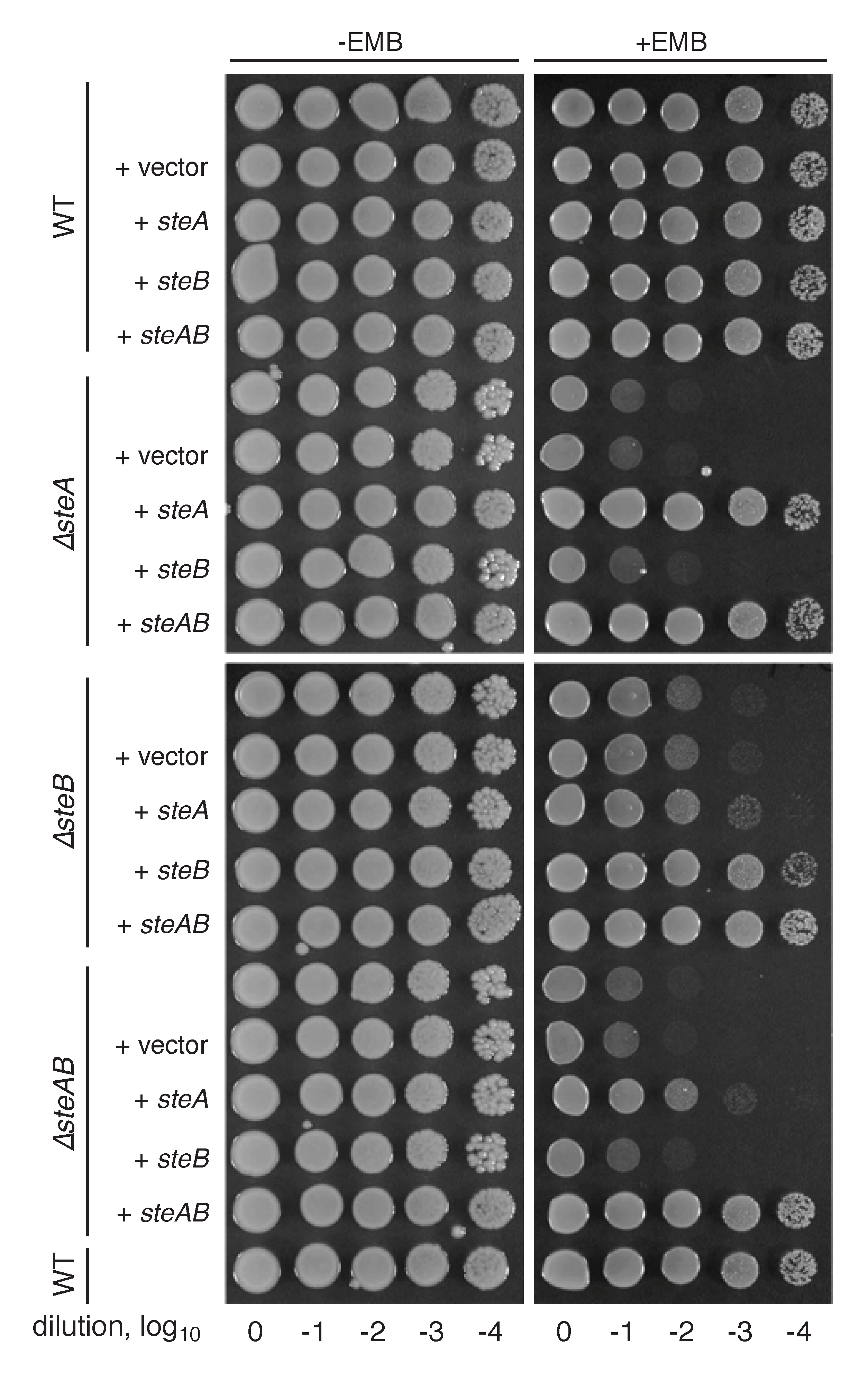

Supplement: S5 Fig — Spot dilutions of MB001 (WT) and the indicated derivatives: ΔsteA (HL2), ΔsteB (HL6) and ΔsteA ΔsteB (HL4). The control vector (pK-PIM) and constructs encoding steA (pHCL57), steB (pHCL59) and the steAB operon (pHCL58) under the PsteA promoter were integrated in the genome of the indicated strains. Overnight cultures of the indicated strains were normalized to OD600 of 0.5, serially diluted, and spotted (5 μl) onto BHI agar medium with and without 0.75 μg/ml EMB as indicated. Plates were incubated for 30 hours at 30°C and photographed. (TIF) [file pgen.1008284.s011.tif]

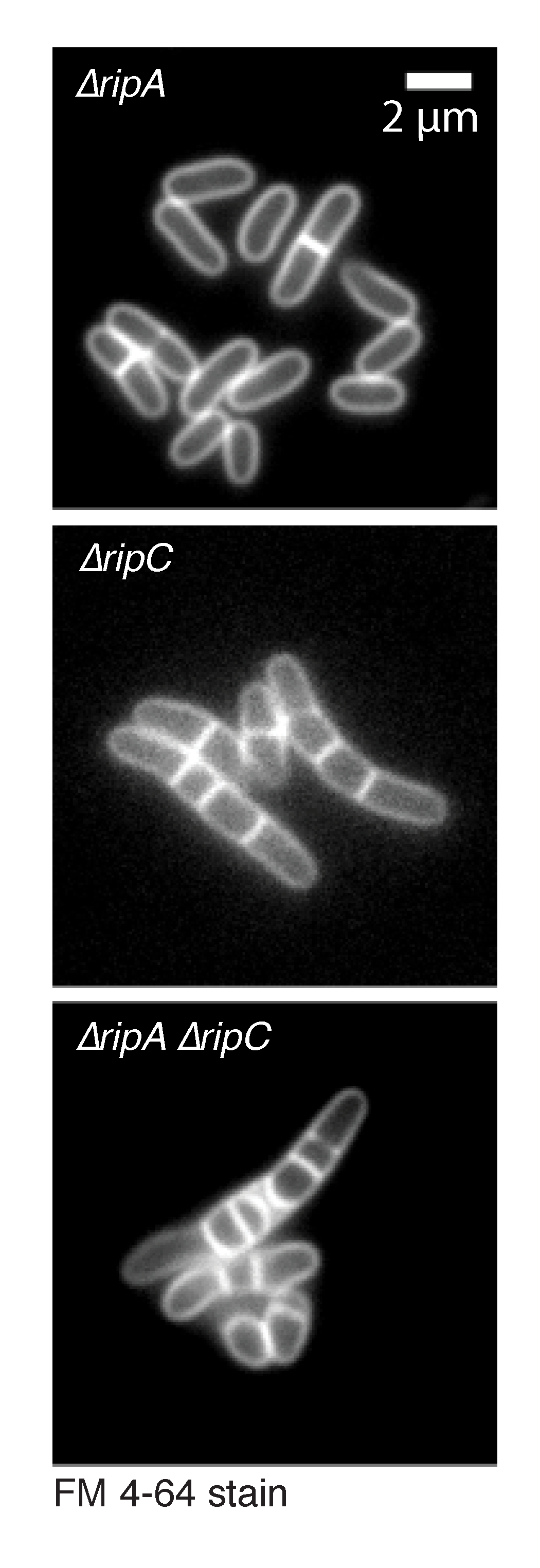

Supplement: S6 Fig — Images of mutants lacking ripA (HL8) or ripC (HL7) or both (HL9). The mutant lacking both genes showed more severe cell separation phenotypes than mutants devoid of only one of those genes, confirming a previously published result [27]. Overnight cultures of the indicated strains were diluted 1:1000 and grown in BHI medium at 30°C. When OD600 of the cultures reached 0.2–0.3, cells were stained with FM 4–64 (1.5 μg/ml) for 5 min, spotted directly on an agarose pad and imaged by fluorescence microscopy. (TIF) [file pgen.1008284.s012.tif]

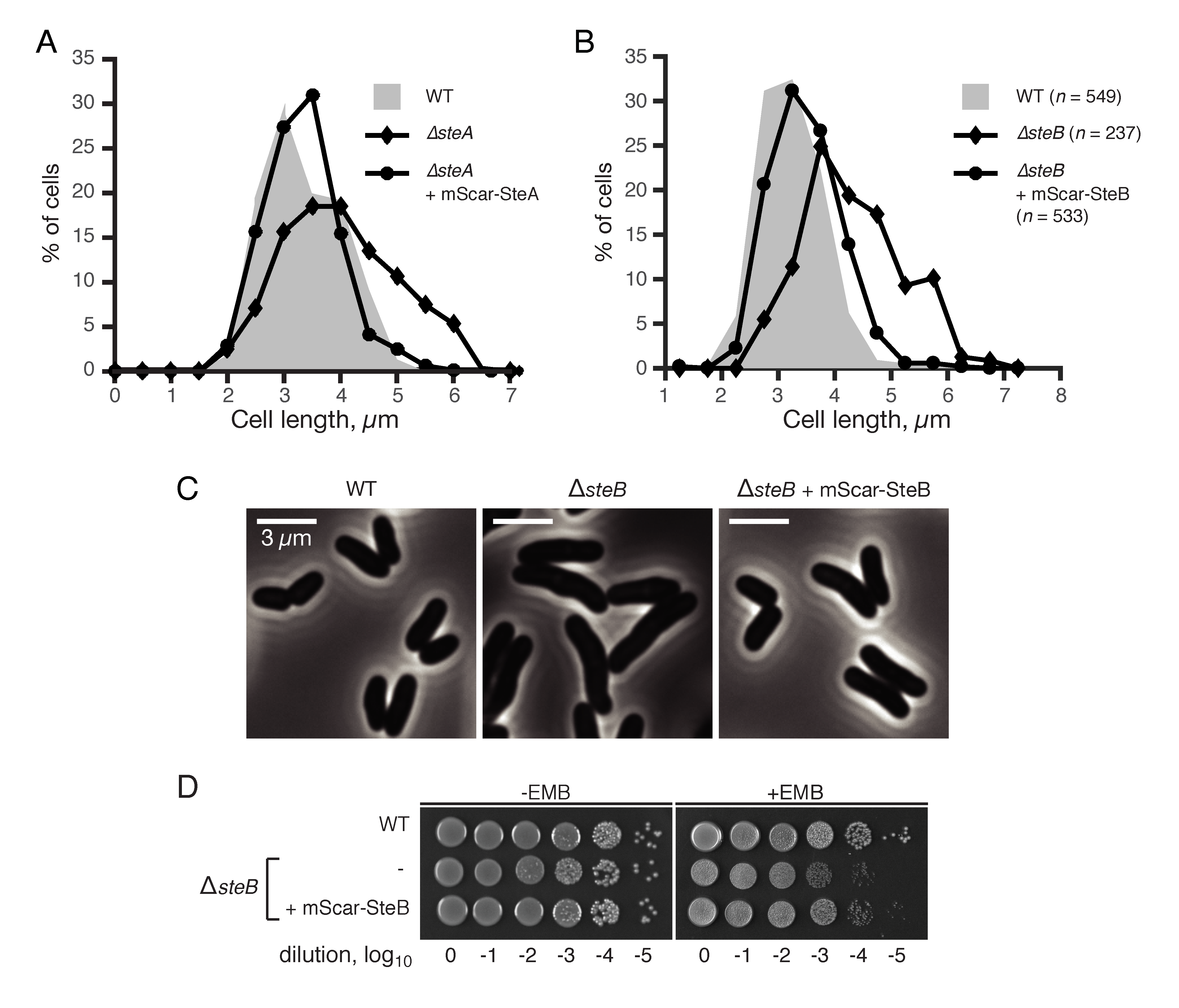

Supplement: S7 Fig — Histograms showing cell length distributions of MB001 (WT) and the indicated derivatives. Both mScar-SteA and mScar-SteB were produced from genome integrated plasmids under PsteA control in the ΔsteA mutant (HL2) and the ΔsteB mutant (HL6), respectively. Overnight cultures were diluted 1:1000 in BHI and grown at 30°C. When the OD600 reached 0.2–0.3, cells were diluted 10-fold and loaded into a CELLASIC ONIX microfluidic device for phase-contrast microscopy. (A & B) Cells were automatically detected from phase-contrast images using Oufti [60]. Cell lengths were calculated from cell outlines using MATLAB. (C) Phase-contrast images of the indicated strains from (B). Scale bars, 3 μm. (D) Overnight cultures of the indicated strains from (B) were normalized to an OD600 of 0.5, serially diluted, and spotted (5 μl) onto BHI agar medium with and without 1 μg/ml EMB as indicated. Plates were incubated for 24 hours at 30°C and photographed. Note that mutants with cell separation defects that form aggregates were vortexed for 3 seconds to resuspend cells before OD600 measurements were taken for normalization. (TIF) [file pgen.1008284.s013.tif]

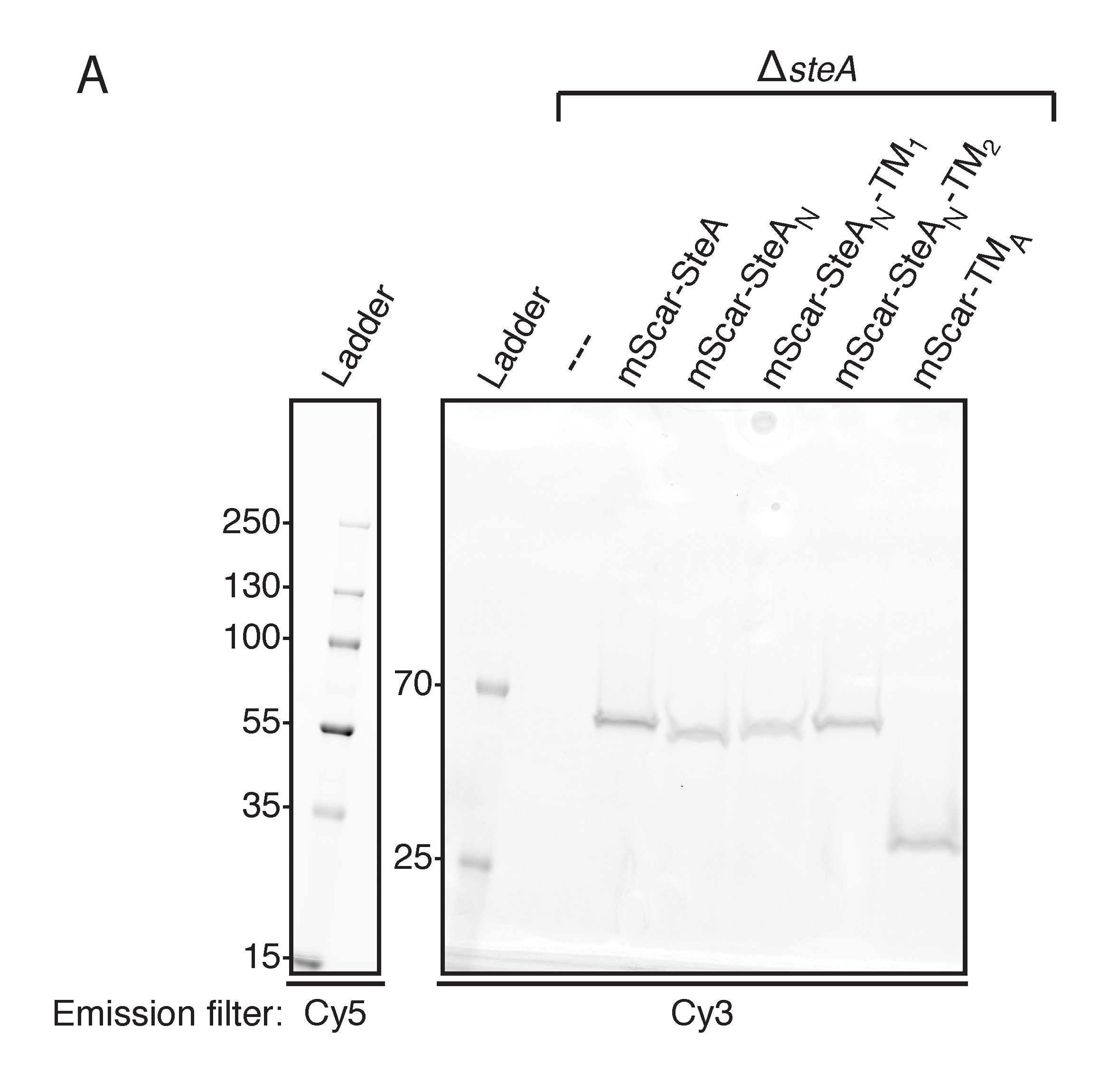

Supplement: S8 Fig — (A) SDS-page gel showing expression levels of the indicated mScar-tagged SteA variants in the ΔsteA mutant. Constructs encoding the indicated mScar-SteA variants under PsteA control were integrated in the genome of the ΔsteA mutant (HL2). Overnight cultures of the indicated strains were diluted to 1:1000 and grown in BHI medium at 30°C. Cells were harvested at OD600 ~ 0.5, resuspended in Buffer A and lysed by lysozyme treatment and sonication. Cell extracts were mixed with SDS loading buffer and resolved on a Pre-cast Criterion TGX gel (Biorad). mScar-fused SteA variants were detected using the Cy3 emission filter, by taking advantage of the intrinsic fluorescence of mScar. Bands in the ladder were revealed using a combination of Cy3 and Cy5 emission filters. Constructs encoding the indicated mScar-SteA variants are as follows: pHCL171: SteA; pHCL172: SteAN; pHCL173: SteAN-TM2; pHCL174: SteAN-TM2; pHCL175: mScar-TMA. (TIF) [file pgen.1008284.s014.tif]

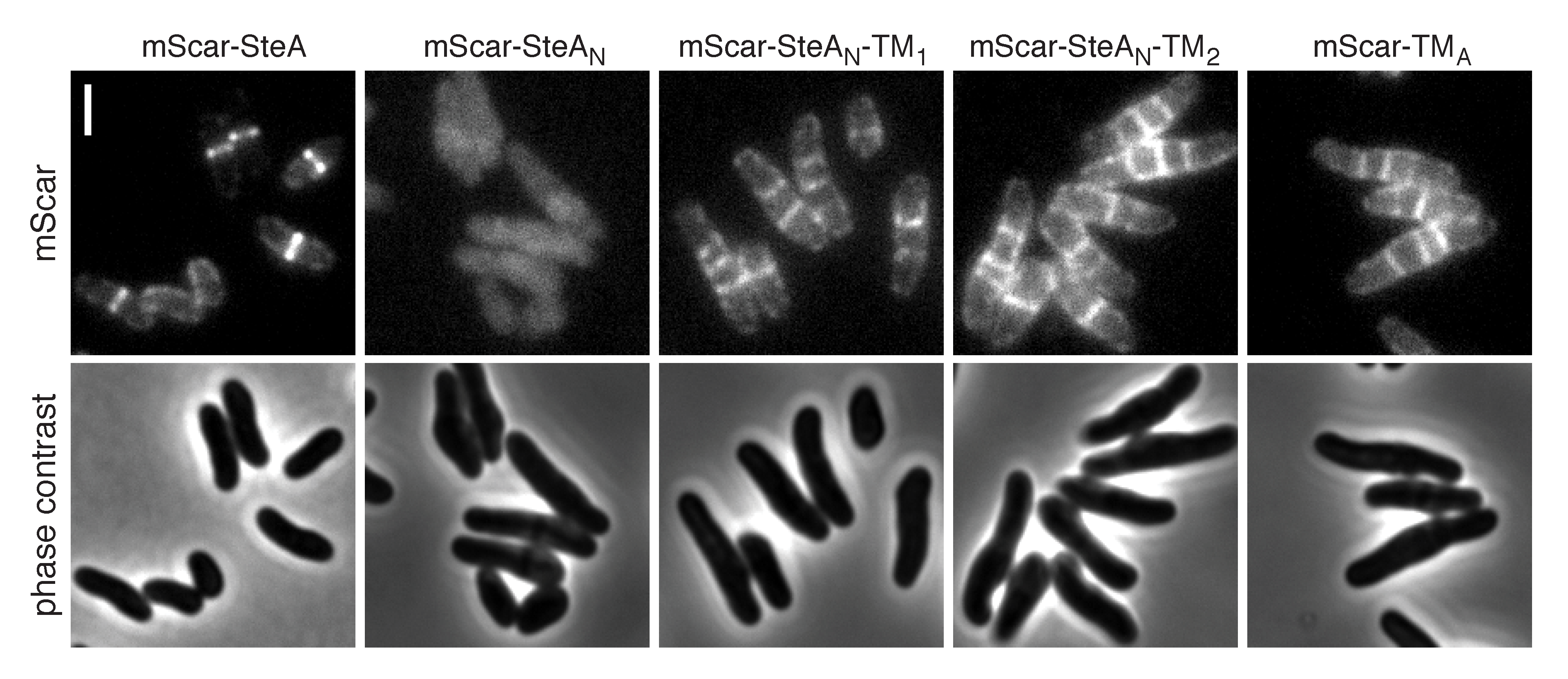

Supplement: S9 Fig — Representative images of the indicated strains from S8 Fig. Overnight cultures were diluted 1:1000 in BHI and grown at 30°C. When the OD600 reached 0.2–0.3, cells were diluted 10-fold and loaded on the CELLASIC ONIX microfluidic device for phase-contrast and fluorescence microscopy. Scale bar, 2 μm. (TIF) [file pgen.1008284.s015.tif]

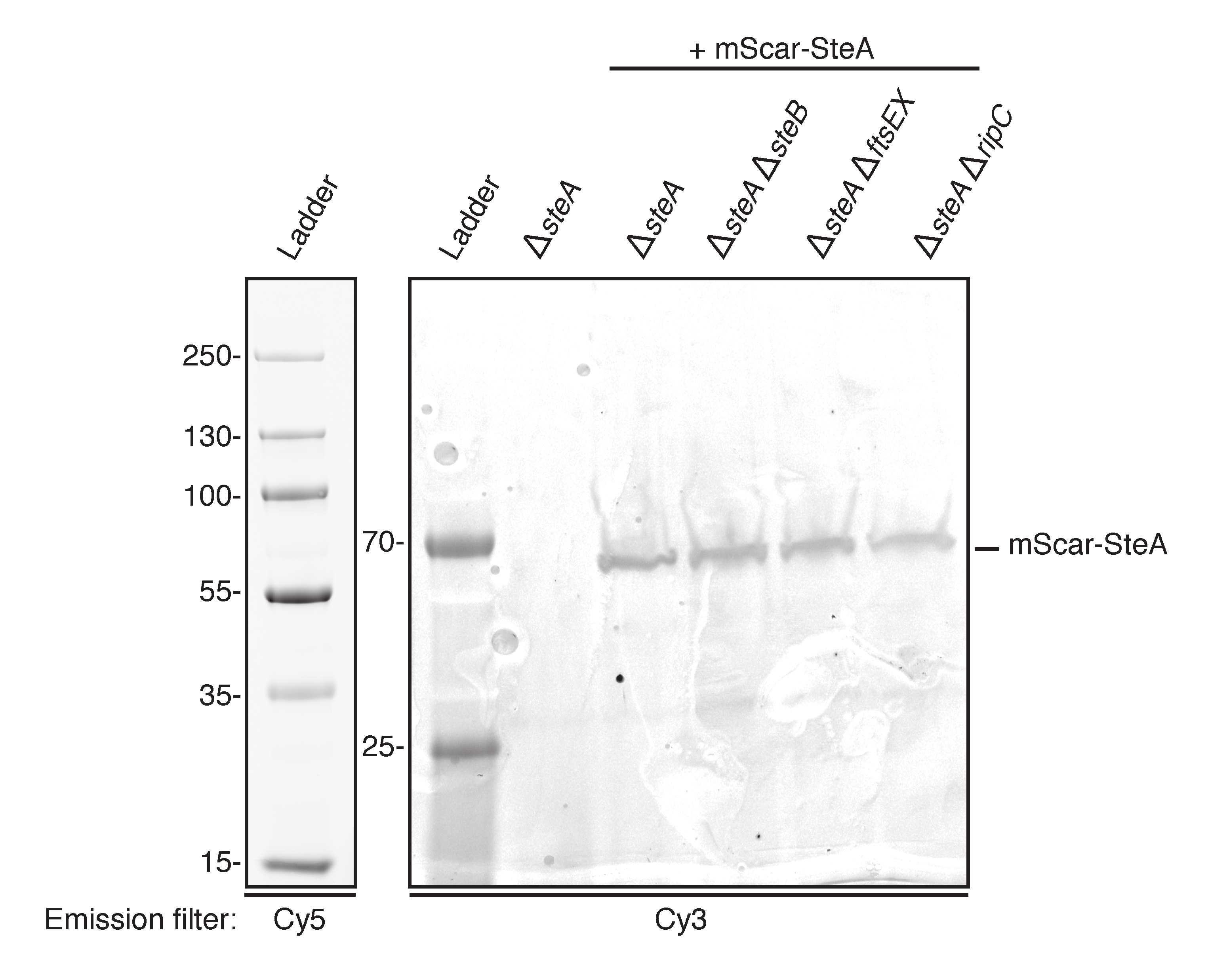

Supplement: S10 Fig — mScar-SteA was produced under PsteA control from pHCL171, which was integrated in the genome of the relevant mutants (HL2: ΔsteA, HL4: ΔsteA ΔsteB, HL10: ΔsteA ΔripC and HL16: ΔsteA ΔftsEX). Overnight cultures of the indicated strains were diluted to 1:1000 and grown in BHI medium at 30°C. Cells were harvested at OD600 ~ 0.5, resuspended in Buffer A and lysed by lysozyme treatment and sonication. Cell extracts were mixed with SDS loading buffer and resolved on a Pre-cast Criterion TGX gel (Biorad). mScar-fused SteA variants were detected using the Cy3 emission filter, by taking advantage of the intrinsic fluorescence of mScar. Bands in the ladder were revealed using a combination of Cy3 and Cy5 emission filters. (TIF) [file pgen.1008284.s016.tif]

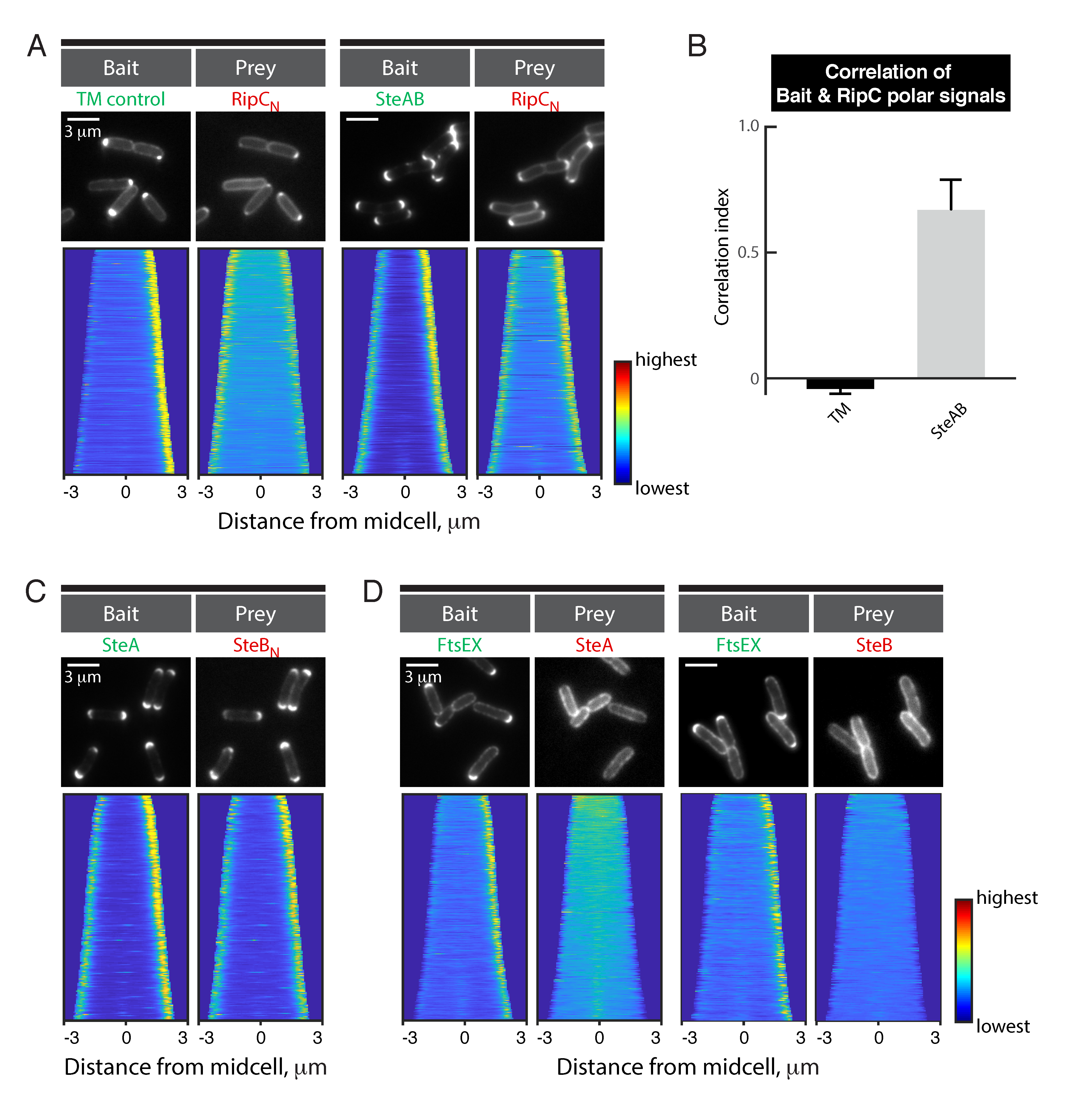

Supplement: S11 Fig — (A, C & D) Top row: representative fluorescence images of TB28 E. coli cells expressing the indicated the bait and prey proteins. Cells were grown for imaging as in Fig 9B. The indicated baits were expressed using the following plasmids: pHCL149 (TM control), pHCL202 (SteA), pHCL204 (SteAB) and pHCL205 (FtsEX). The preys were expressed from genome integrated plasmids (pHCL194: SteB, pHCL196: SteBN, pHCL214: SteA and pHCL225: RipCN). Bottom rows: demographs showing fluorescence distribution of the corresponding preys and baits at the population level. Single-cell fluorescence quantification was performed using Oufti [60]. Cells were oriented using a custom-written MATLAB script such that the cell pole with the higher bait fluorescence was located on the right of the demograph. At least 250 cells were used to generate each demograph. (B) Quantification of fluorescence correlation between the bait-prey pairs at the cell pole was performed as described in Fig 10A. (TIF) [file pgen.1008284.s017.tif]

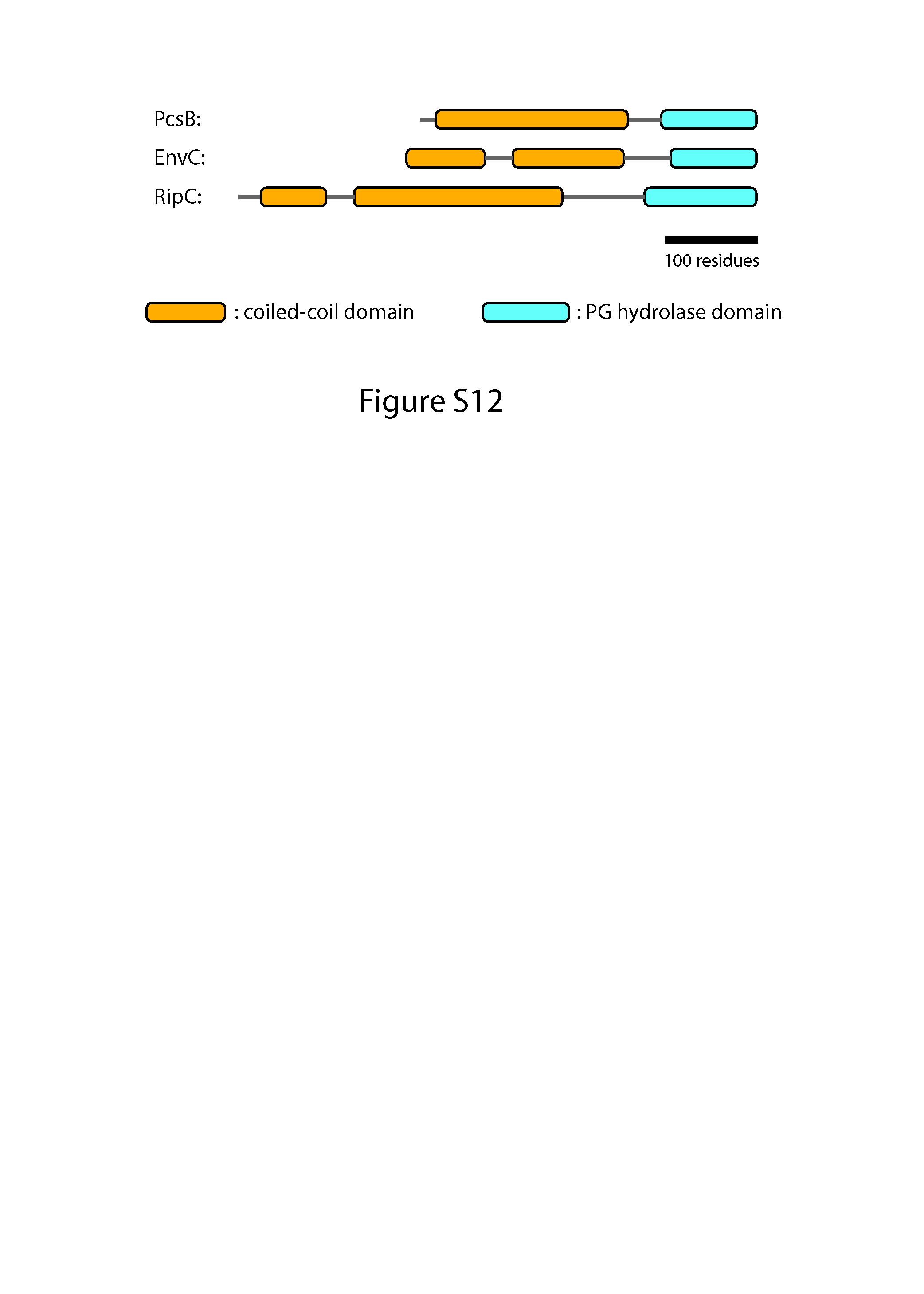

Supplement: S12 Fig — Scaled domain representations of PcsB (spd_2043), EnvC (b3613) and RipC (cgp_1735). The coiled coil region of PcsB was identified from a solved X-ray crystal structure of the protein (PDB ID: 4CGK) [65]. Coiled coil regions RipC and EnvC were predicted using the algorithm of Lupas et al. [66]. The corresponding PG hydrolase domain of each protein was identified using Pfam [67]. The signal sequence of each protein was identified using the Phobius algorithm [68] and excluded from the cartoon representation. (TIFF) [file pgen.1008284.s018.tiff]
